# Reducing Peptidoglycan Crosslinking by Chemical Modulator Reverts β‐lactam Resistance in Methicillin‐Resistant *Staphylococcus aureus*

**DOI:** 10.1002/advs.202400858

**Published:** 2024-05-15

**Authors:** Ji‐Hoon Kim, Yunmi Lee, Inseo Kim, JuOae Chang, Subin Hong, Na Kyung Lee, David Shum, Seongeun Baek, Wooseong Kim, Soojin Jang, Wonsik Lee

**Affiliations:** ^1^ School of Pharmacy Sungkyunkwan University Suwon 16419 Republic of Korea; ^2^ Antibacterial Resistance Laboratory Institut Pasteur Korea Seongnam 13488 Republic of Korea; ^3^ Screening Discovery Platform Institut Pasteur Korea Seongnam 13488 Republic of Korea; ^4^ College of Pharmacy Graduate School of Pharmaceutical Sciences Ewha Womans University Seoul 03760 Republic of Korea

**Keywords:** β‐lactam resistance, c‐di‐AMP, celastrol, peptidoglycan, *Staphylococcus aureus*

## Abstract

Small molecule can be utilized to restore the effectiveness of existing major classes of antibiotics against antibiotic‐resistant bacteria. In this study, it is demonstrated that celastrol, a natural compound, can modify the bacterial cell wall and subsequently render bacteria more suceptible to β‐lactam antibiotics. It is shown that celastrol leads to incomplete cell wall crosslinking by modulating levels of c‐di‐AMP, a secondary messenger, in methicillin‐resistant *Staphylococcus aureus* (MRSA). This mechanism enables celastrol to act as a potentiator, effectively rendering MRSA susceptible to a range of penicillins and cephalosporins. Restoration of in vivo susceptibility of MRSA to methicillin is also demonstrated using a sepsis animal model by co‐administering methicillin along with celastrol at a much lower amount than that of methicillin. The results suggest a novel approach for developing potentiators for major classes of antibiotics by exploring molecules that re‐program metabolic pathways to reverse β‐lactam‐resistant strains to susceptible strains.

## Graphical abstract

This study provides insight into the mechanism of celastrol as a potentiator of β‐lactams against methicillin‐resistant Staphylococcus aureus (MRSA). Celastrol reduces the cellular c‐di‐AMP level by activating GdpP and increases muropeptides with shorter glycine branches that cannot be processed by PBP2a of MRSA, making MRSA susceptible to β‐lactams. The findings suggest that modulation of c‐di‐AMP can be a therapeutic target for enhancing β‐lactams.

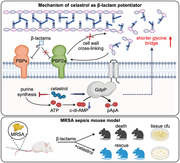

## 1 Introduction

The prevalence of bacterial infections caused by antibiotic‐resistant strains has significantly increased in recent decades, posing a serious burden on public health and the global economy. In the COVID‐19 pandemic era, multiple major antibiotics including β‐lactams were prophylactically prescribed to hospitalized COVID‐19 patients, which resulted in a significant increase in the occurrence of antibiotic‐resistant bacteria.^[^ ^1^ ^]^ Moreover, the number of deaths caused by antibiotic‐resistant bacteria including methicillin‐resistant *Staphylococcus aureus* (MRSA),^[^ ^2^ ^]^ vancomycin‐resistant Enterococci (VRE),^[^ ^3^ ^]^ and carbapenem‐resistant *Acinetobacter baumannii* (CRAB),^[^ ^4^ ^]^ is expected to surpass the toll of cancer in 2050.^[^ ^5^ ^]^ Despite the pressing demand for antibiotics with novel mechanisms of action capable of inactivating resistant bacteria, the number of compounds under development in the clinic has significantly declined.^[^ ^6^ ^]^

β‐lactam antibiotics remain one of the most widely used classes of antimicrobial agents for treating bacterial infections around the world. The β‐lactam antibiotics, which inhibit the transpeptidation of peptidoglycan (PG) in bacterial wall biogenesis, include penicillins, cephalosporins, monobactams, and carbapenems.^[^ ^7^ ^]^ This class has a clinically well‐established toxicity profile and is relatively inexpensive to produce because the synthesis process of β‐lactams is well‐established.^[^ ^8^ ^]^ However, bacterial infections caused by β‐lactam‐resistant bacteria are becoming increasingly common and are a main cause of severe infection‐associated diseases.^[^ ^9^ ^]^ In most cases, the β‐lactam resistance arises through the expression of β‐lactamases that degrade β‐lactams. To overcome the resistance by β‐lactamase, β‐lactamase inhibitors, such as clavulanic acid, have been developed and successfully applied in the clinic.^[^ ^8^, ^10^ ^]^ However, MRSA also carries a resistant gene cassette containing *mecA*, which encodes the β‐lactam resistant penicillin‐binding protein, PBP2a. PBP2a exhibits low affinity for most β‐lactams, allowing it to maintain PG crosslinking even in the presence of β‐lactams. To overcome β‐lactam resistance by PBP2a of MRSA, newer generations of β‐lactams such as ceftaroline and ceftobiprole have been developed. However, resistance against these antibiotics has already emerged in the clinic.^[^ ^11^ ^]^

In this study, we demonstrate that celastrol, a natural compound, can restore β‐lactam susceptibility in MRSA through remodeling the PG, by which co‐treatment of β‐lactam and celastrol increases the potency of β‐lactams against MRSA. We show that celastrol directly reduces c‐di‐AMP levels in *S. aureus*. Based on our biochemical analyses, we propose that a reduced level of cellular c‐di‐AMP restores susceptibility to β‐lactams by modifying PG crosslinking rendering PBP2a of MRSA incapable of processing PG formation. This novel mechanism of celastrol, exerting significant effects on cell wall crosslinking, can be largely applicable to the development of potentiators for β‐lactams, and our work underscores the utility of combination therapies for targeting infections caused by β‐lactam‐resistant bacteria.

## 2 Results

### 2.1 Celastrol Restores the Susceptibility of MRSA Against β‐lactams

**Figure 1:**
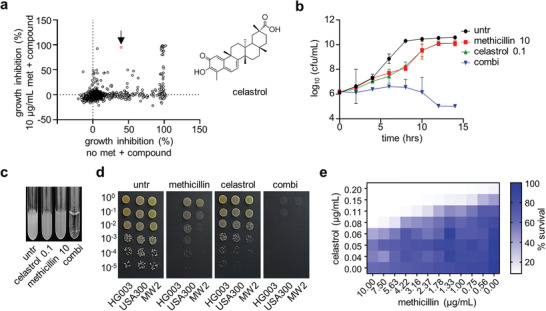
Celastrol leads to overcome a methicillin resistance in MRSA. a) Growth inhibition in the presence of methicillin was plotted against growth inhibition in the absence of methicillin for 2321 compounds used for screening. The arrow indicates celastrol and its structure is presented on the right. b) Cultures of USA300 were treated with antibiotics (10 µg mL^−1^ methicillin, 0.1 µg mL^−1^ celastrol, or combination (combi) of the two); CFUs were monitored for 14 h. Each data point is represented as the mean ± standard deviation of three biological replicates. c) Images of cultures were taken at 14 h post‐treatment. d) Spot dilution assay of serial dilutions of bacterial cultures was performed on TSB agar containing 0.4 µg mL^−1^ celastrol, 4 µg mL^−1^ methicillin, or combination (combi) of the two as indicated. Plates were incubated at 30 °C for 16 h before being imaged. The images are representative of three biological replicates. e) Checkerboard microdilution assay of methicillin and celastrol was performed against USA300. Data are representative of at least three biological replicates.

The resistance mechanisms of MRSA against β‐lactams, including methicillin, depend on the hydrolysis of the β‐lactam ring or the production of alternative PBPs such as PBP2a with lower affinity to β‐lactams.^[^ ^12^ ^]^ In this study, we designed a cell‐based assay to identify compounds that can target the resistance mechanism of MRSA or can drive modification of the crosslinking of PG and thereby potentiate the inhibition of transpeptidation by β‐lactams. We tested compounds in two growth conditions of *S. aureus* USA300: no methicillin or 10 µg mL^−1^ methicillin was added to all wells. Comparing these two growth conditions allowed us to filter out compounds that demonstrated β‐lactam‐independent growth inhibition of MRSA. We screened 2321 small molecules comprising bioactive compounds at 10 µm in duplicates at each growth condition in 384‐well plates (**Figure** **1**a). Plates were incubated for 22 h at 30 °C and bacterial growth was measured by optical density at 600 nm. We set cutoffs based on percent growth inhibition compared to the control not exposed to any drug or compound by each compound and found 23 lethal compounds (class IV, Figure S1, Supporting Information). Interestingly, we found that 73 compounds lose their antibacterial potency in the presence of methicillin. Within class II compounds that kill MRSA only in the presence of methicillin, we identified two potential re‐sensitizers, among which the most potent one, celastrol, is an NRPS (non‐ribosomal peptide synthetase)‐derived natural product (Figure 1a).

First reported in 1981, celastrol is a natural compound isolated from the root extracts of *Tripterygium wilfordii*.^[^ ^13^ ^]^ It is a pentacyclic nortriterpen quinone that belongs to the family of quinone methides^[^ ^14^ ^]^ and was shown to play a role as a Nur77 inducer that alleviates inflammation and induces autophagy.^[^ ^15^ ^]^ Moreover, preclinical studies have demonstrated its efficacy as a treatment option for obesity^[^ ^16^ ^]^ and its potent anticancer properties against various types of tumors, such as liver, cancer, and leukemia,^[^ ^14b^ ^]^ providing promising results for its clinical application. Also, recently, celastrol appeared as a hit in a compound screen to identify inhibitors of the proton motive force (PMF) of the bacterial membrane.^[^ ^17^ ^]^ However, the impact of its intracellular targeting in the bacterial cell has never been characterized. We further characterized the activity of celastrol and determined its mechanism of action, which enables further development as a possible potentiator of β‐lactam antibiotics.

**Figure 2:**
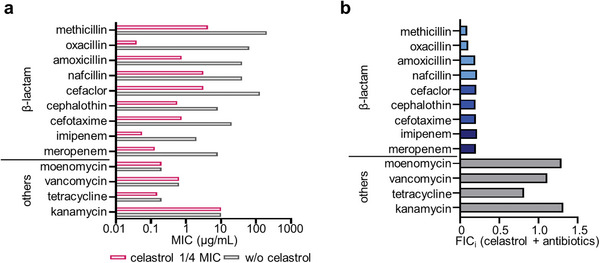
Celastrol potentiates specifically β‐lactam against MRSA. a) The changes in MICs for β‐lactams were tested in the presence of 0.15 µg mL^−1^ (1/4 MIC) celastrol. b) FICi values for antibiotics are presented. FIC_i_  =  FIC*_antibiotics_* + FIC*_celastrol_*, (FIC  =  [X]/MIC, X = MIC in combination). Data are representative of at least three biological replicates.

Co‐treatment of methicillin and celastrol was highly potent against MRSA USA300 with complete killing at 0.1 µg mL^−1^ celastrol and 10 µg mL^−1^ methicillin, a much lower concentration than that required when methicillin is treated alone (Figure 1b). Separate treatment with 10 µg mL^−1^ methicillin or 0.1 µg mL^−1^ celastrol did not show significant growth inhibition. In combination, however, co‐treatment of the same doses of methicillin and celastrol demonstrated an outstanding antimicrobial activity in killing MRSA cultures at an exponential phase (Figure 1c). This synergistic effect was also validated by spot dilution assay (Figure 1d) and was further confirmed by a checkerboard assay (Figure 1e). According to the results of the checkerboard assay, an even lower amount of celastrol (0.08 µg mL^−1^) was also sufficient to render MRSA susceptible to methicillin; the minimum inhibitory concentration (MIC) of USA300 against methicillin decreased 40‐fold in the presence of celastrol. However, since the phenotype could be due to non‐specific, β‐lactam‐independent potentiation mechanisms, we performed the same analyses with other classes of antibiotics to exclude this possibility. We found that only β‐lactams including penicillins and third and fourth generation cephalosporins showed the synergy effect while other classes such as aminoglycosides or quinolones did not show any detectable synergy (**Figures** **2**a and S2, Supporting Information). Furthermore, to examine possible synergy with non‐β‐lactam antibiotics targeting PG synthesis, we tested moenomycin and vancomycin that inhibit the glycosyltransferase (GT) activity but not transpeptidase, and as shown in Figure 2a,b, no synergy was found. These results suggest that celastrol affects the crosslinking of PG that β‐lactams target. Next, we tested the effectiveness of celastrol in combination with methicillin and oxacillin against other MRSA strains including MW2 and three clinical isolates. All tested MRSA strains in the presence of a low dose of celastrol (0.2–1 µg mL^−1^) became susceptible to β‐lactams, while no synergy was found with vancomycin and moenomycin (Figures S3 and S4, Supporting Information). This data further confirms that the mechanism of potentiation by celastrol might involve the transpeptidation activity of MRSA's PBPs.

### 2.2 Celastrol Potentiates β‐lactams Against MRSA through Mechanisms Independent of the Proton Motive Force Inhibition

**Figure 3:**
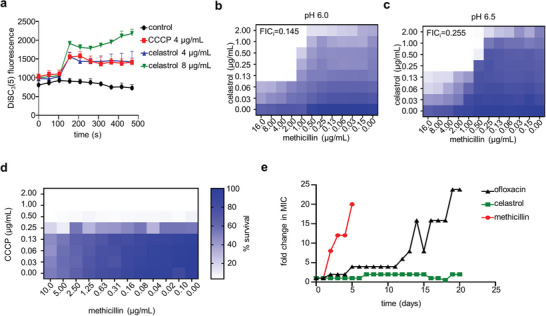
Celastrol potentiates β‐lactams independently of proton motive force inhibition. a) Cultures of USA300 at the exponential phase were stained with DiSC_3_(5), and fluorescence was measured using a microplate reader at 620 nm after treatment with celastrol or CCCP. b,c) The synergistic effect of celastrol and β‐lactam antibiotics was tested in MHB at b) pH 6.0 and c) 6.5 using checkerboard assay. d) A checkerboard assay was conducted to verify the synergistic effect of CCCP and methicillin. FICi  =  FIC*_methicillin_* + FIC*_CCCP_*, (FIC  =  [X]/MIC, X = MIC in combination). FICi ≤ 0.5 means synergistic. The results are representative of at least three independent experiments. e) Acquisition of spontaneous resistance was determined during serial passaging in the presence of sub‐MIC concentrations of antibiotics or celastrol. The fold changes in MICs at each passage were plotted. The figure is representative of three independent experiments.

Since we found that celastrol alone shows antibacterial potency at a high concentration (Figure 1e), we measured celastrol MICs against a panel of bacterial strains. As shown in Figure S5a (Supporting Information), celastrol showed antibacterial efficacy against Gram‐positive pathogens: vancomycin‐resistant (or susceptible) *Enterococcus faecalis*, methicillin‐susceptible *S. aureus* (MSSA) HG003, and *Bacillus subtilis* (MIC = 0.15–0.6 µg mL^−1^), while being ineffective against all tested Gram‐negative strains: *Pseudomonas aeruginosa*, *A. baumannii*, and *Escherichia coli*. We reasoned that the inactivity against Gram‐negative bacteria might be due to the presence of the outer membrane barrier. To test this possibility, we examined the efficacy of celastrol in *lpxC* deficient *A. baumannii* which has a weaker lipopolysaccharide (LPS) layer.^[^ ^18^ ^]^ As expected, the Δ*lpxC* mutant showed susceptibility to celastrol and its MIC was measured as 0.04 µg mL^−1^. To further confirm the penetration of celastrol, we treated Gram‐negative bacteria with colistin which primarily attaches to the LPS and drives pore formation and found that the co‐treatment of celastrol with colistin showed a strong synergistic effect in both *E. coli* and *A. baumannii* (Figure S6a–c, Supporting Information). As recent study,^[^ ^17^ ^]^ we also found that celastrol treatment of USA300 resulted in a significant increase in the signal from the DiSC_3_(5) dye, comparable to that produced by the known reference compound carbonyl cyanide m‐chlorophenyl hydrazine (CCCP) also demonstrating PMF perturbation (**Figure** **3**a; Figure S5b, Supporting Information). This PMF perturbation by celastrol was also observed in Δ*lpxC* mutant (Figure S6e,f, Supporting Information). We then examined the effect of the PMF inhibition on the β‐lactam potentiation. Given that PMF inhibition is influenced by proton concentration, we tested the MICs of celastrol against USA300 under acidic conditions, and as shown in Figure S5c (Supporting Information), the MIC of celastrol increased at pH 6.5 and 6.0 compared to that at pH 7.3. However, the synergy between celastrol and methicillin remained similar in all tested pH conditions (Figure 3b,c). Furthermore, CCCP did not show a synergistic effect with methicillin (Figure 3d). Importantly, these results suggest that the mechanisms of the synergistic effect of celastrol are independent of PMF interference during the penetration.

We next examined the frequency of resistant mutant formation by performing a serial passage every 24 h for 21 days under exposure to three compounds: methicillin, ofloxacin, and celastrol. A MRSA strain USA300 was treated with sub‐inhibitory concentrations of each compound, and every day we measured changes in the MICs of the serially passaged cultures. As shown in Figure 3e, further development of methicillin resistance in USA300 was observed starting from the second day, with a rapid increase in resistance (up to 20x MIC) further on. In the case of ofloxacin, the MIC also increased 24‐fold at 20 days. However, we found no increase in the MIC against celastrol during the entire test period. This low frequency of occurrence of resistance to celastrol again suggests that the mechanisms of action of celastrol may involve multiple cellular targets in addition to the PMF disruption. Furthermore, since the synergistic effect of celastrol with methicillin was observed at a lower concentration than the MIC of celastrol alone, another independent mechanism of action of celastrol might be involved in the synergy with β‐lactams.

### 2.3 Celastrol Converts MRSA to MSSA by Lowering Intracellular c‐di‐AMP

**Figure 4:**
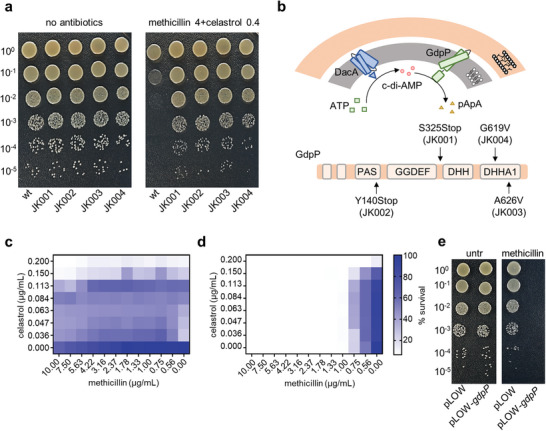
Celastrol modulates cellular c‐di‐AMP and the reduction in c‐di‐AMP levels confers β‐lactam susceptibility. a) Spot dilution assay was performed by plating serial dilutions of the USA300 parent strain and four isolated resistant mutants on TSB agar containing 0.4 µg mL^−1^ celastrol and 4 µg mL^−1^ methicillin as indicated. Plates were incubated at 30 °C for 16 h before being imaged. The images are representative of three biological replicates. b) Schematic of c‐di‐AMP synthesis and degradation in *S. aureus* illustrates that DacA generates c‐di‐AMP from ATP and then GdpP hydrolyses c‐di‐AMP into 5′‐pApA. The schematic below represents altered residues within four domains of GdpP in four resistant strains. c,d) Checkerboard microdilution assays of methicillin and celastrol were performed against USA300 harboring USA300 c) Δ*gdpP* and d) pLOW‐*gdpP*. e) Serial dilutions of USA300 harboring pLOW‐*gdpP* or empty plasmid were plated on TSB agar containing 2 µg mL^−1^ methicillin or no antibiotic for spot dilution assay. Plates were incubated at 30 °C for 16 h before being imaged. Data are representative of at least three biological replicates (c–e).

Although celastrol may disrupt the PMF of the plasma membrane when it penetrates, another independent mechanism of action of celastrol might be involved during the co‐treatment with β‐lactams since the synergistic effect of celastrol with methicillin was observed at a lower concentration than the MIC of celastrol alone. To identify potential cellular targets of celastrol, we raised spontaneous mutants of USA300 under the co‐treatment condition, 0.4 µg mL^−1^ celastrol and 4 µg mL^−1^ methicillin. We isolated several mutants, and the resistant phenotype of the selected mutants was validated in the presence of both methicillin and celastrol (Figure S7a, Supporting Information). They show no observable growth defects in the absence of antibiotics (**Figure** **4**a). Also, these mutants have no resistance to PMF disruption by celastrol, suggesting that the mutations acquired are related to cellular targets other than PMF (Figure S7b,c, Supporting Information). To identify the genes that conferred resistance, we performed whole genome sequencing of the mutants, and among the identified mutations, all the mutant strains shared the inactivation of *gdpP* (SAUSA300_0014) as shown in Figure 4b. GdpP has phosphodiesterase activity utilizing the cyclic dinucleotide c‐di‐AMP produced by DacA, a diadenylate cyclase in *S. aureus*, as a substrate. Therefore, the level of cellular c‐di‐AMP, a regulatory messenger, is controlled by the activities of DacA and GdpP.^[^ ^19^ ^]^ Cellular c‐di‐AMP has been implicated in controlling the integrity of the cell envelope.^[^ ^20^ ^]^ To confirm the role of *gdpP* in the mechanisms of celastrol, we performed a checkerboard assay using USA300 Δ*gdpP* and found no synergy, as expected (Figure 4c). Since resistance acquired by the loss of function of *gdpP* suggests that elevated levels of c‐di‐AMP can lead to β‐lactam resistance, we reasoned that lowering cellular c‐di‐AMP levels could result in β‐lactam susceptibility. To test this, we overexpressed *gdpP* in USA300 to reduce cellular c‐di‐AMP, and as shown in Figure 4d,e, the overexpression of *gdpP* led to USA300 being highly susceptible to methicillin. This data is consistent with the results of a *gdpP* deficient strain.

**Figure 5:**
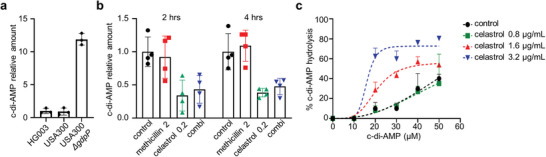
Celastrol drives β‐lactam susceptibility through draining c‐di‐AMP by activation of GdpP. a) Intracellular c‐di‐AMP concentrations of HG003, USA300, USA300 Δ*gdpP* were measured by LC‐ToF/MS. Each point represents mean ± standard deviation from three biological replicates. b) Intracellular c‐di‐AMP concentration of USA300 was determined after antibiotic treatment as indicated. Cultures were incubated at 30 °C for 2 or 4 h before being analyzed. c) Assessment of GdpP enzyme activity was performed in an assay buffer containing 0–50 µm c‐di‐AMP and 1 µm GdpP in the presence of celastrol as indicated. Reactions were quenched after 30 min at 37 °C, and the amount of hydrolyzed c‐di‐AMP was measured by LC‐ToF/MS. Each data point represents mean ± standard deviation from three replicates.

Next, to further confirm the role of c‐di‐AMP in the mechanism of celastrol, we measured intracellular c‐di‐AMP in the Δ*gdpP* mutant. As expected, intracellular c‐di‐AMP levels in the Δ*gdpP* strain significantly increased, which suggests a crucial role of GdpP in c‐di‐AMP production in *S. aureus* (**Figure** **5**a). Next, to examine the effect of celastrol on the levels of c‐di‐AMP, we treated USA300 with 0.2 µg mL^−1^ of celastrol, 2 µg mL^−1^ methicillin, or both. As shown in Figure 5b, upon treatment with celastrol, the cellular c‐di‐AMP of USA300 decreased significantly. Then, we hypothesized that the reduction of levels of c‐di‐AMP that confers methicillin susceptibility could be achieved by either inhibition of DacA or enhancement of GdpP. First, to test the possibility of celastrol as an antagonist for DacA, we performed a DacA enzyme assay with purified DacA of USA300 in the presence of celastrol. However, as shown in Figure S8 (Supporting Information), DacA activity remained unchanged in the presence of celastrol. Next, we tested if celastrol enhances the enzymatic activity of GdpP. With purified GdpP, we performed a GdpP enzyme assay which measures the conversion of c‐di‐AMP to 5`‐pApA. As shown in Figure 5c, the yield of substrate conversion by GdpP in the presence of celastrol was elevated over fourfold compared to when celastrol was absent, and such elevation was observed in a dose‐dependent manner. We observed that a higher concentration of celastrol is required to activate GdpP in vitro compared to the concentration needed for β‐lactam potentiation. This could be attributed to non‐optimal conditions of the enzyme assay we employed or the potential involvement of other cofactors. Together, our results demonstrate the role of celastrol as a negative modulator of cellular c‐di‐AMP levels through activation of GdpP in *S. aureus*.

### 2.4 Celastrol Drives Modification of PG Crosslinking through Regulation of c‐di‐AMP

**Figure 6:**
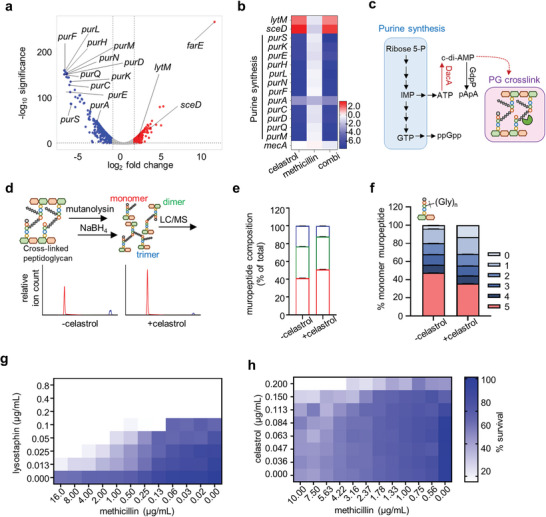
Reduction in c‐di‐AMP levels by celastrol alters PG crosslinking and increases amounts of shortened glycine bridges. a,b) Transcriptomic changes of USA300 were analyzed after 2 h treatment with celastrol, methicillin, or both. Genes with significantly altered expressions are presented as a volcano plot generated using GraphPad 9.0. The cut‐off for *p* value was 0.05, while that for log_2_ fold change was ±1.5. Genes related to purine biosynthesis are marked and indicated with arrows. Overexpressed genes are in red, while repressed genes are in blue (a). Log_2_ fold changes in gene expression of USA300 treated with antibacterials relative to the DMSO‐treated control are expressed as a heat map (b). c) Pathways involved in purine biosynthesis, c‐di‐AMP generation, and PG crosslinking are represented as a schematic. d–f), Muropeptides of PG were analyzed after treatment with celastrol for 4 h at 37 °C. The procedure for analysis of crosslinked muropeptide species is represented as a schematic (d upper), and representative extracted ion chromatograms show the crosslinking products from cells treated with celastrol: monomers (red), dimers (green), and trimers (blue). Data are representative of three independent experiments (d lower). The areas of each peak were integrated and quantified, and percentages of the three muropeptides are presented: monomers (red), dimers (green), and trimers (blue) (e). Upon treatment with celastrol, the alteration in muropeptides with different lengths of glycine bridges were compared (f). The means and standard deviations are from three biological replicates. g,h) Checkerboard microdilution assay of lysostaphin and celastrol was performed against USA300 (g) and that of methicillin and celastrol was performed against Tn::*lytM* USA300 (h). Data are representative of at least three biological replicates.

Next, we performed a transcriptomic analysis of USA300 treated with celastrol to provide possible mechanisms related to the remodeling of cell wall biogenesis and other mechanisms, such as induction of drug efflux pumps, that could possibly trigger resistance against celastrol. We treated *S. aureus* USA300 with methicillin or celastrol alone or in combination for 2 h. As shown in **Figure** **6**a, the expressions of 121 genes were significantly increased (log_2_FC >1.5) while those of 190 genes were decreased (log_2_FC <1.5). Interestingly, we found that *farE* involved in the efflux of hydrophobic toxic substances, such as antimicrobics and antimicrobial fatty acids,^[^ ^21^ ^]^ was found to be strongly induced in the presence of celastrol (>2000‐fold change in both co‐treatment and celastrol alone). This hike in *farE* expression suggests that celastrol penetrates to the inside of the cell as demonstrated above and has intracellular targets. We also found other genes, such as *lytM*, related to PG maintenance and autolysis, were significantly induced upon treatment with celastrol. Interestingly, we found a strong deactivation of the entire purine synthesis pathway, which is known to interplay with cellular c‐di‐AMP. The genes that were significantly altered were confirmed by quantitative real‐time PCR (Figure S9, Supporting Information). However, we found that PBP2a (*mecA*) expression was not significantly altered, suggesting that celastrol acts in a PBP2a‐independent mode (Figure 6b).

c‐di‐AMP as a second messenger is implicated in the stress response or maintenance of cell wall homeostasis through controlling PG crosslinking in many pathogenic bacteria including *S. aureus*.^[^ ^19^ ^]^ Interestingly, among the induced genes, we found *lytM*, encoding an endopeptidase that cleaves between the third and the fourth peptide bond of the pentaglycine bridge of *S. aureus* PG (Figure 6c). Then, we hypothesized that the reduced c‐di‐AMP through elevated GdpP activity by celastrol might result in the modification of PG crosslinking. The changes in PG crosslinking could convert MRSA to be susceptible to β‐lactams. To test this, we treated USA300 with a low dose of celastrol (0.1 µg mL^−1^) for 4 h and analyzed the muropeptide patterns of purified PG. We identified monomers, dimers, and trimers of muropeptides by their molecular weights, and as shown in Figure 6d,e, we found that the proportion of monomers was significantly increased upon celastrol treatment while that of multimeric species (dimers and trimers) was reduced compared to the untreated group, suggesting reduced crosslinking in PG. Furthermore, we found that muropeptides with full‐length pentaglycine, (Gly)_5_, were reduced while muropeptides with a shorter glycine bridge (1‐4) were increased (Figure 6f). We note that PBP2a of MRSA is capable of the utilization of only muropeptides with (Gly)_5_ and partially (Gly)_3_, while native PBPs can use shorter ones,^[^ ^22^ ^]^ which implies that muropeptides with shorter (Gly)_n_ generated during celastrol treatment would not be processed by the PBP2a of MRSA.

Next, since lysostaphin cleaves PG just as LytM does, we hypothesized that methicillin might have a synergistic effect with lysostaphin. Indeed, as shown in Figure 6g, we found that the MICs of methicillin against USA300 dropped in the presence of lysostaphin. These results suggest that celastrol drives modification of crosslinking in PG, which may be driven by hydrolases including LytM. To further characterize the involvement of LytM in modification of the crosslinks, we tested the synergy of celastrol and methicillin against USA300 Tn*::lytM* and found an abolishment of synergy (Figure 6h), suggesting that LytM plays a crucial role in this mechanism of action, although this result would not exclude the potential involvement of other hydrolases. *lytM* is one of many genes found in the regulon of a two‐component system, WalKR. While *lytM* was significantly upregulated, other genes, such as *isaA* and *ssaA*, in the WalKR regulon remained unchanged in the presence of celastrol, suggesting that celastrol may induce a response independent of the WalKR two‐component system (Table S1, Supporting Information). Next, to observe possible morphological changes in MRSA by celastrol, we performed transmission electron microscopy and did not find significant changes in either the cocci shape or septum (Figure S10, Supporting Information), which suggests that celastrol has a negligible impact on the division machinery of *S. aureus*, since an imbalance or defect in division machinery would result in observable septal abnormality.^[^ ^23^ ^]^

### 2.5 Celastrol Co‐Treatment Shows a Protective Effect In Vivo in a Mouse Sepsis Model

**Figure 7:**
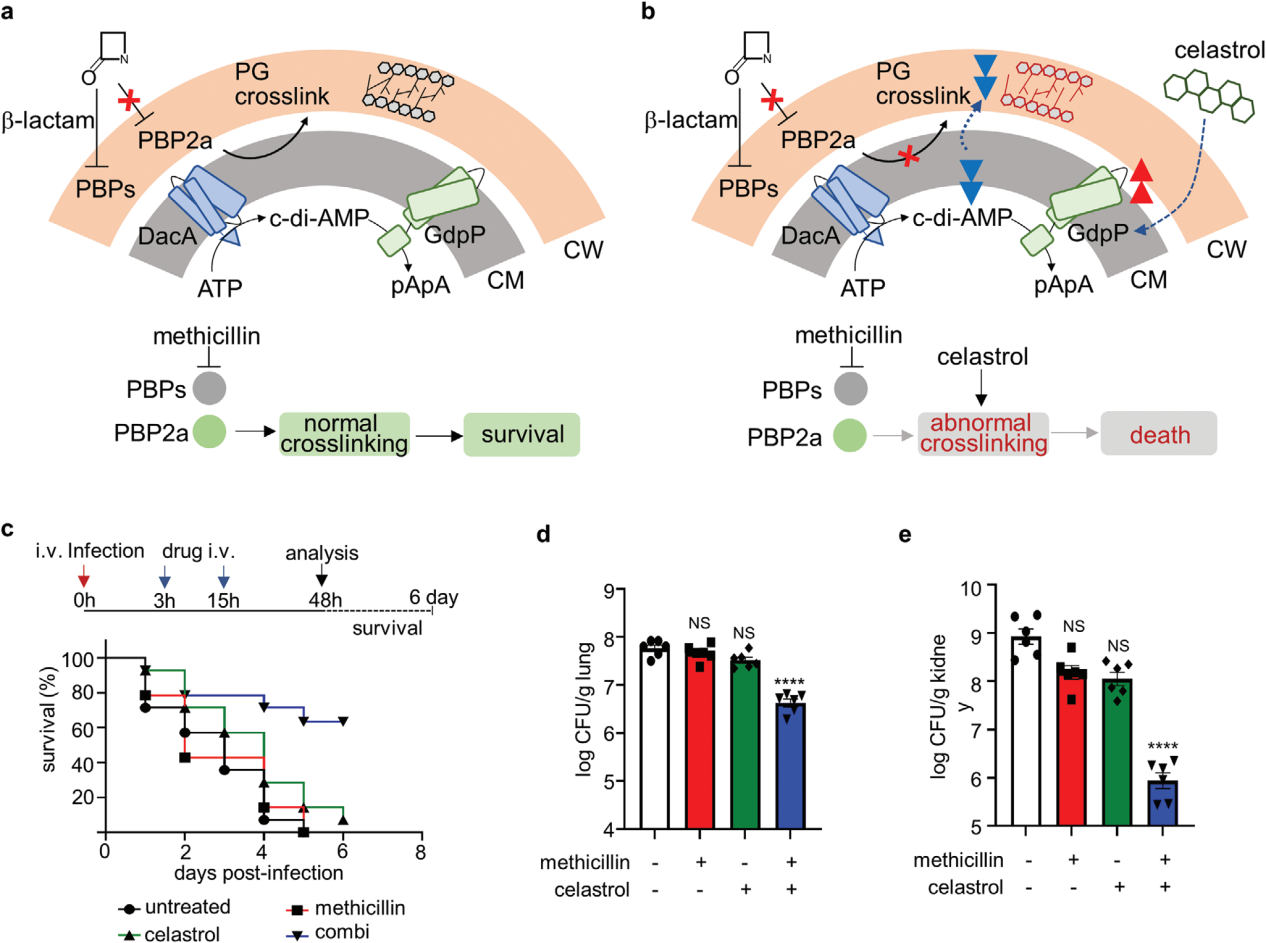
Combination of methicillin and celastrol demonstrates efficacy against MRSA in vivo. a,b) Model of celastrol activity as a modulator of PG crosslinking. In MRSA, celastrol penetrates the plasma membrane, after which celastrol enhances the activity of GdpP. GdpP activation leads to a decrease in cellular c‐di‐AMP, and the change signals to modify the crosslinking of PG, which converts MRSA to a β‐lactam susceptible strain. The model shows in the presence of methicillin (a) or methicillin and celastrol (b). CM, cytoplasmic membrane; CW, cell wall. c–e) BALB/c mice were given a sublethal dose of USA300 (1 × 10^8^ CFUs/mouse and were administered with DMSO, methicillin, celastrol, or their combination (n = 14 per group) by intravenous injection as indicated (c). c) Survival curves of the animals in each treatment group are shown during 6 days post infection. d,e) Bacterial loads in the lung (d) and kidneys (e) are shown at 2 days post infection. Error bars represent mean ± standard error of mean for biological replicates.

Finally, we propose that penetrated celastrol reduces cellular c‐di‐AMP through activating GdpP, thereby both reducing the PG crosslinks and altering the length of the glycine bridge of muropeptides to limit processing by the PBP2a of MRSA (**Figure** **7**a,b). Given the promising mechanism of action of celastrol, we examined its potential as a therapeutic agent in a mouse sepsis model. BALB/c mice were infected intravenously with USA300 at a dose that leads to death in 50% of injected mice in 48 h. Three hours post‐infection, the first dose of the drug was administered: methicillin 36 mg kg^−1^, celastrol 0.002 mg kg^−1^, and a combination of both. The same dose of drugs was injected 12 h after the first drug administration. Celastrol is known to demonstrate unfavorable pharmacokinetics such as relatively low solubility and high toxicity (>1.5 mg kg^−1^).^[^ ^24^ ^]^ However, we used a concentration much lower than the toxic concentration (750x lower) in our combination treatment, and this concentration of celastrol allows further clinical applications. We found no observable side effects in the treated animals as demonstrated through the weight profile (Figure S11, Supporting Information). Celastrol retains relatively low permeability, but this property may be advantageous to treat bacteremia which is usually treated by intravenous drug administration. As shown in Figure 7c, the animal group co‐treated with celastrol and methicillin survived, while methicillin or celastrol alone did not effectively protect the infected animals. The co‐treatment showed an outstanding efficacy, causing more than 2.2 log reduction of CFUs in the lung (Figure 7d) and 3 log reduction in the kidneys at 2 days post infection (Figure 7e). Importantly, we note here that the dose of methicillin used in the combination group for the treatment of MRSA USA300 was the amount that suffices to treat MSSA infection.

## 3 Discussion

In this study, we report that celastrol, a natural product, can act as a potentiator that reprograms β‐lactam‐resistant MRSA to susceptible MSSA. We have shown that celastrol has an activity of modification of the crosslinking in PG through the reduction of cellular c‐di‐AMP. We have shown that celastrol directly activates the activity of GdpP that hydrolyzes c‐di‐AMP thereby reducing the intracellular level of c‐di‐AMP. We further showed that this change reduces the crosslinking of PG and the length of pentaglycine bridge linked to the stem peptide. Therefore, we have established that the mechanism of action of celastrol that potentiates β‐lactams is through the modification of PG crosslinking, which is independent of PMF inhibition by celastrol. Finally, we showed that as a potentiator for β‐lactams, celastrol is a promising therapeutic candidate; co‐treatment with methicillin and a small amount of celastrol (over 10 000‐fold less than methicillin) was potent against MRSA in a mouse sepsis model.

It has been demonstrated that the native *S. aureus* PBPs can process cell wall precursors having different glycine‐branch lengths (Gly_5_, Gly_3_, or Gly_1_), while PBP2a of MRSA exhibits significant preference for PG strands with a complete pentaglycine branch.^[^ ^22^ ^]^ Especially, PBP2a cannot crosslink PG with monoglycine. In *S. aureus*, FemA adds the second and third glycines of the pentaglycine branch, and FemB adds the fourth and fifth. The *femA* or *femB* deficient *S. aureus* strains are still viable suggesting that the native PBPs can use Gly_1_ and Gly_3_ PG as crosslinking substrates. However, these deficient mutants of MRSA show increased susceptibility against β‐lactams, which also implies that PBP2a of MRSA is not capable of processing the PG substrate with a shorter glycine bridge. Therefore, because of this preference of PBP2a, FemA and FemB have been implicated as targets for small molecules of β‐lactam potentiators. Along with these previous observations, our work with celastrol further emphasizes the importance of understanding PBP2a's substrate preference.

As previously reported, the inactivation of purine production such as in Δ*purF* resulted in a significant decrease in cellular c‐di‐AMP levels in *S. aureus*, indicating a cross‐talk between the purine pathway and the c‐di‐AMP flow.^[^ ^25^ ^]^ We also found that the treatment with celastrol which results in a decrease of cellular c‐di‐AMP significantly suppressed the purine pathway, suggesting a reciprocal relationship between these two pathways. Therefore, regarding the cellular c‐di‐AMP level, treatment with celastrol not only activates GdpP but also deactivates purine biosynthesis, further exacerbating the decrease in c‐di‐AMP levels and its downstream targets, including PG crosslinking. Recently, Yuan and colleagues showed that celastrol alone suppresses multiple metabolic pathways in *S. aureus* including purine biosynthesis; and they showed, using in vitro assay, that celastrol has an affinity to Δ1‐pyrroline‐5‐carboxylate dehydrogenase (P5CDH) required for proline metabolism.^[^ ^26^ ^]^ However, the mechanism of celastrol that we established here in the presence of methicillin, is a potentiator mechanism independent of P5CDH. Although we demonstrate the molecular mechanism of celastrol as a β‐lactam potentiator, it remains to be determined through additional research which potential regulators orchestrate and sense cellular c‐di‐AMP and signal to induce genes required for the modification of PG crosslinking.

## 4 Conclusion

The rise in the occurrence of β‐lactam resistant bacteria among many multidrug‐resistant pathogens has led to the emergence of infections that are hard to treat. Combination therapies as demonstrated in this study show promise in combating resistance and reducing the dose of β‐lactams used to treat infection. We demonstrate cellular c‐di‐AMP as a potential therapeutic target in a combination treatment to reduce β‐lactam resistance. We envision that our findings could be applicable in discovering novel antibiotic potentiator scaffolds and identifying unlethal metabolic pathways whose modulation can be explored to reduce antibiotic resistance.

## 5 Experimental Section

### Materials and Bacterial Strains

Unless otherwise indicated, all chemicals and reagents were purchased from Sigma–Aldrich. Celastrol (#C0869, Batch Number 0000219458) was also purchased from Sigma–Aldrich. Restriction enzymes were purchased from New England Biolabs. *E. coli* strains were grown with shaking at 37 °C in lysogeny broth (LB) or on LB agar. *S. aureus* strains were grown at 30 °C or 37 °C in Tryptic Soy Broth (TSB) or on TSB agar (TSA). Culture media were purchased from BD Difco. A list of bacterial strains used in the study can be found in Table S2 (Supporting Information).

### Compound Screen

To discover candidates for β‐lactam re‐sensitizers, compound screening was conducted under the following conditions: the same set of test compounds at a 10 µm final concentration was added to two different 384‐well plates: 1) a plate containing 10 µg mL^−1^ methicillin and 2) a plate not containing methicillin in a final volume of 40 µL of TSB. *S. aureus* USA300 was then inoculated to both plates at an OD_600_ of 0.002 and was cultured at 30 °C for 22 h. The OD_600_ was measured by a microplate reader (BioTek, Synergy HTX). The cutoff was defined as a case where growth inhibition was more than 75% compared to the control not exposed to any drug or compound.

### Minimal Inhibitory Concentration (MIC) Assay and Checkerboard Assay

The MICs of the compounds used in this study were determined by the standard microdilution method. Briefly, a single colony of bacteria on an agar plate was inoculated in broth and incubated to log phase at 37 °C with shaking. Subsequently, the compounds were diluted using a serial dilution method in a 96‐well microplate and the bacterial culture was added to each well in a final MHB volume of 150 µL. The plate was incubated with shaking at 37 °C for 16–18 h and OD_600_ was measured using a spectrophotometer (BioTek, Synergy HTX). The antimicrobial effect of combination treatment of celastrol and antibiotics was determined through a checkerboard assay.^[^ ^27^ ^]^ Each well consisted of a combination of different concentrations of celastrol and antibiotics to be tested by serially diluting the compounds. Bacteria of interest were inoculated in the plate, and after incubating at 37 °C for 18 h, the OD_600_ was determined with a spectrophotometer (BioTek, Synergy HTX).

### Membrane Potential Perturbation Assay

The membrane potential was measured by DiSC_3_(5) dye, a membrane‐permeable fluorescent dye.^[^ ^28^ ^]^ Exponential phase *S. aureus* USA300 was collected by centrifugation, and then washed three times in buffer containing 50 mm HEPES, 300 mm KCl, and 0.1% glucose (pH 7.2). After a final wash, the pellet was re‐suspended in the same buffer and adjusted to an OD_600_ of 0.085. DiSC_3_(5) was added to a final concentration of 1 µm and incubated at RT for 7 min, followed by 7 min at 37 °C. Fluorescence was monitored up to 500 s after the addition of the appropriate amount of drug. CCCP was added as a positive control. The fluorescence signal was measured at an excitation wavelength of 620 nm and emission wavelength of 670 nm at 37 °C.

### Spot Dilution Assay

TSA plates supplemented with celastrol, methicillin, or both were prepared. An overnight culture of bacteria was diluted in fresh TSB to an OD_600_ of 0.05 and cultured until reaching an OD_600_ of 1. Several tenfold serial dilutions of the bacterial culture were spotted onto the agar plates and incubated overnight at 30 °C until being imaged by Nikon D3100 AF‐S 18–55 mm.

### Generation of Spontaneous Mutants and Whole Genome Sequencing

To generate mutants that survive in co‐treatment conditions with celastrol and methicillin, *S. aureus* USA300 was cultured on TSA plates supplemented with 4 µg mL^−1^ of methicillin and 0.4 µg mL^−1^ of celastrol. After incubating at 30 °C overnight, colonies that survived in the combination treatment condition were picked from the plates. For whole genome sequencing (WGS) of mutants, bacterial DNA was extracted using a Qiagen DNeasy kit according to the manufacturer's instructions. To determine the genetic changes in mutants compared to the wild‐type USA300, WGS analysis was performed at Macrogen (Seoul, Republic of Korea) with Illumina Hiseq2000. A total read of samples ranged from 13.7 to 15.0 million. Paired‐end read sequencing was performed with read lengths of 151 bp. Genome assembly and identification of genetic alterations, including SNPs, deletions, and insertions, were performed using Geneious v11.0.9.

### Induction of Resistant Mutants by Serial Passaging

Serial passaging was conducted to identify whether antibiotic resistance was induced.^[^ ^29^ ^]^ A 96‐well plate with serially diluted antibiotics was prepared, with 1x MIC included in the range of concentrations. Ofloxacin served as a positive control. After 24 h incubation at 30 °C, the second highest compound concentration at which growth of the bacterial culture (OD_600_ ≥ 0.5) was observed was chosen for the next day's MIC assay. Then all cultures were stored at −80 °C. This serial passaging was repeated daily for 20 days. In the case of methicillin, the percentage of DMSO in the broth was becoming too high as MIC increased, so serial passaging was stopped before 20 days.

### In Vivo Infection Assay with a Mouse Sepsis Model

All in vivo animal experimental procedures were approved by the Sungkyunkwan University Animal Ethical Committee in accordance with the guidelines of the Korean Animal Protection Law (SKKUIACUC2022‐12‐35‐1). Eight‐week‐old male BALB/c mice (Orient, Korea) were used in this study. The mice were randomly divided into four groups: 1) vehicle, 2) methicillin, 3) celastrol, or 4) methicillin plus celastrol. The mice were injected intravenously with *S. aureus* USA300 (1 × 10^8^ CFU/mouse) to induce sepsis. After 3 h of infection, the first drug injection of methicillin (36 mg kg^−1^), celastrol (0.002 mg kg^−1^), or saline was intravenously administered; the second drug injection was conducted 12 h after the first drug injection. Organs (lung and kidneys) were harvested at 2 days post‐infection and homogenized in PBS. The organ homogenates were plated on TSA and incubated at 30˚C overnight for determination of bacterial colony forming units (CFU).

### Construction of the USA300 pLOW‐*gdpP* and *ΔgdpP* Strains

For generation of *S. aureus* pLOW‐*gdpP*, the *gdpP* sequence (SAUSA300_0014) of *S. aureus* USA300 was cloned using *E. coli* (DH5α) competent cells into the pLOW plasmid^[^ ^30^ ^]^ using *SalI*(G/TCGAC) and *BamHI*(G/GATCC) sites, and the resulting plasmid was transformed into *S. aureus* RN4220 electro‐competent cells with Gene Pulser Xcell Electroporation System (Bio‐Rad) using a 0.2 cm Gene Pulse Cuvette (Bio‐Rad). Bacteriophage (ϕ11)‐mediated transduction was performed from RN4220 to the donor strain USA300. For generation of *S. aureus* Δ*gdpP*, bacteriophage Φ11 was used to transduce genes from RN4220 Δ*gdpP* to USA300. The Δ*gdpP* deletion of USA300 was confirmed by PCR.

### GdpP and DacA Protein Purification

The full‐length *gdpP* gene was amplified by PCR from the genomic DNA of *S. aureus*. Subsequently the PAS, GGDEF, and DHH/DHHA1 domain of *gdpP* was cloned into the expression vector pET‐28a between the *NheI* and *XhoI* restriction sites. The plasmid harboring the DNA construct and His6 tag was transformed into *E. coli* BL21(DE3). For protein expression, 1 L of BL21 was incubated to an OD_600_ of 0.8, and 0.8 mm isopropyl‐d‐thiogalactopyranoside was added to induce protein expression. The bacterial culture was incubated at 16 °C for 12 h and then centrifuged at 6000 *g* for 15 min. After removing the supernatant, the cells were resuspended using lysis buffer (50 mm Tris pH 8.0, 150 mm NaCl, 5% glycerol, 0.1% β‐mercaptoethanol, 100 µg mL^−1^ DNase, 10 mm MgCl_2_, 250 µg mL^−1^ lysozyme, 1 mM PMSF) and incubated at 4 °C for 30 min. Subsequently, the bacteria cells were lysed by sonication. After centrifugation at 12 000 rpm at 4 °C for 30 min, the supernatant was collected and incubated with Ni‐NTA beads for 2 h at 4 °C. The solution was washed with W1 buffer (lysis buffer with 20 mm imidazole) and W2 (lysis buffer with 50 mm imidazole). The protein was eluted by filtering with elution buffer (50 mm Tris pH 8.0, 150 mm NaCl, 5% glycerol, 500 mm imidazole).

For the expression of DacA protein, the *dacA* gene (SAUSA300_2113) was amplified by PCR from the genomic DNA of *S. aureus*. Subsequently the catalytic domain of *dacA* was cloned into the expression vector pET‐28a between the *NheI* and *HindIII* restriction sites. The plasmid harboring the DNA construct and His6 tag was transformed into *E. coli* BL21(DE3). For protein expression, 1 L of BL21 was incubated to an OD_600_ of 0.5, and 0.5 mm isopropyl‐d‐thiogalactopyranoside was added to induce protein expression. The bacterial culture was incubated at 37 °C for 3 h, and then centrifuged at 6,000 *g* for 15 min. After removing the supernatant, the cells were resuspended using a lysis buffer (50 mm Tris pH 7.5, 500 mm NaCl, 100 µg mL^−1^ DNase, 10 mm MgCl_2_, 250 µg mL^−1^ lysozyme, 1 mm PMSF) and incubated at 4 °C for 30 min. Subsequently, the bacteria cells were lysed by sonication. After centrifugation at 12 000 rpm at 4 °C for 30 min, the supernatant was collected and incubated with Ni‐NTA beads for 2 h at 4 °C. The solution was washed with wash buffer (lysis buffer with 10 mm imidazole). The protein was eluted by filtering with elution buffer (50 mm Tris pH 7.5, 200 mm NaCl, 500 mm imidazole). High purity proteins were pooled together after SDS‐PAGE analysis. The protein was flash frozen and stored at −80 °C.

### GdpP and DacA Enzymatic Activity Assay

Enzymatic activity of GdpP protein was measured by incubating purified protein with c‐di‐AMP in reaction buffer (20 mm KCl, 50 mm Tris pH 8.5 and 0.1 mm MnCl_2_) at 37 °C. An equal volume of 0.1 m EDTA (pH 8) was added to stop the reaction and the mixture was incubated at 95 °C for 3 min. For DacA activity assay, the enzyme was incubated with ATP in reaction buffer (40 mm HEPES (pH 7.1), 0.7 NaCl, 1 mm MnCl_2_).^[^ ^31^ ^]^ Concentrations of c‐di‐AMP were detected by LC‐ToF mass spectrometry (MS) consisting of an Agilent Mass 6230 time‐of‐flight (ToF) coupled to an Agilent 1290 liquid chromatography (LC) system. The column used for analysis was a 3 × 150 mm, 2.7 µm Poroshell 120 HILIC column (Agilent Technologies), and the solvent conditions were as follows: the mobile phase consisted of solvent A (ddH_2_O with 0.2% formic acid) and solvent B (acetonitrile with 0.2% formic acid). The gradient condition used was as follows: 0–2 min, 85% B; 3–5 min, 80% B; 6–7 min, 75% B; 8–9 min, 70% B; 10–11.1 min, 50% B; 11.1–14 min, 20% B; 14.1–24 min, 5% B followed by a 10 min re‐equilibration period at 85% B at a flow rate of 0.4 mL min^−1^.

### Measurement of Cellular c‐di‐AMP in USA300 by LC‐ToF/MS

USA300 was incubated in TSB supplemented with celastrol, methicillin, or both at 30 °C. Then, the culture was added to the mixture of MeOH/acetonitrile/H_2_O (40:40:20) for metabolic quenching after which mechanical lysis was performed with 0.07–0.11 mm glass beads using a homogenizer (Precellys, Bertin Technologies).^[^ ^32^ ^]^ The cell lysates were filtered through a sterile 0.2 µm microcentrifuge filter, and protein concentration was determined using a BCA Protein Assay kit (Thermo Scientific). Extracted metabolites were separated using a 3 × 150 mm, 2.7 µm Poroshell 120 HILIC column (Agilent Technologies). The mobile phase consisted of solvent A (ddH_2_O with 0.2% formic acid) and solvent B (acetonitrile with 0.2% formic acid). The gradient condition used was as follows: 0–2 min, 85% B; 3–5 min, 80% B; 6–7 min, 75% B; 8–9 min, 70% B; 10–11.1 min, 50% B; 11.1–14 min, 20% B; 14.1–24 min, 5% B followed by a 10 min re‐equilibration period at 85% B at a flow rate of 0.4 mL min^−1^. An Agilent Mass 6230 time‐of‐flight (ToF) coupled to an Agilent 1290 liquid chromatography (LC) system was used to identify metabolites. The detected ions were considered metabolites based on mass‐retention time identifiers for masses showing the expected distribution of accompanying isotopomers. Metabolites were identified using the Agilent MassHunter Profinder software with a mass tolerance of ± 0.005 Da.

### Muropeptide Analysis by LC‐ToF/MS

Muropeptide was analyzed as previously described.^[^ ^33^ ^]^ *S. aureus* USA300 was inoculated in TSB and grown at 30 °C with aeration overnight. Overnight bacterial culture was diluted to OD_600_ of 0.02 in 100 mL fresh TSB with celastrol and grown for 4 h at 37 °C. Bacterial cells were harvested from 100 mL of each culture. The pellet was washed with PBS and resuspended in 4% SDS. The cells were boiled at 100 °C for 30 min and the cell walls were centrifuged at 17 000 g for 10 min. The walls were washed at least five times with distilled water. The suspension was concentrated by centrifugation at 17 000 g for 10 min. The pellet was treated with 100 mm Tris‐HCl (pH 7.5) containing α‐amylase at 37 °C for 2 h to remove any bacterial glycogen present. The suspension was then mixed with DNase and RNase and incubated for 1 h at 37 °C. Finally, the suspension was treated with trypsin for 18 h at 37 °C and centrifuged at 17 000 g for 10 min. The pellet was resuspended with 1% SDS and boiled at 100 °C for 20 min for inactivation of the enzymes. The cell walls were centrifuged at 17 000 g for 10 min and thoroughly washed with distilled water after which 1 m HCl was added and incubated for 4 h at 37 °C to release WTA. The suspension was centrifuged at 17 000 g for 10 min and washed with distilled water twice until the pH dropped to 5–6. The pellet was treated with 25 mm phosphate buffer containing mutanolysin for 20 h at 37 °C. The suspension was subsequently boiled for 7 min at 100 °C to inactivate mutanolysin. The suspension was centrifuged for 10 min at 17 000 g to remove insoluble materials. Finally, the supernatant was collected and stored at −80 °C. For LC‐MS analysis, the sample was mixed with 0.5 m borax with 10 mg mL^−1^ sodium borohydride for 30 min at RT. Phosphoric acid was then added to the mixture to adjust the pH to 2–3.

The samples were analyzed by LC‐MS on an Agilent 6520 TOF mass spectrometer equipped with ESI‐MS operating in a positive mode. Muropeptide products were separated on a Waters Symmetry Shield RP18 column (5 µm, 3.9 × 150 mm) with the following conditions: flow rate = 0.5 mL min^−1^, 100% solvent A (H_2_O, 0.1% formic acid) for 5 min followed by a linear gradient of solvent B (acetonitrile, 0.1% formic acid) from 0% to 20% over 40 min. Molecular ions for the target muropeptide fragments were extracted from the total ion chromatogram.

### Transcriptomic Analysis of MRSA

Total RNA was extracted from *S. aureus* USA300 using the Qiagen RNeasy kit. Celastrol (0.05 µg mL^−1^) and methicillin (25 µg mL^−1^) were added to the bacterial suspension of OD_600_ of 0.3 and the cultures were incubated at 37 °C for 2 h. Cells were pelleted and the RNA isolation was conducted according to the kit protocol. cDNA library construction and RNA‐seq were performed by Macrogen (Seoul, Republic of Korea). Briefly, the RNA was converted into a cDNA library using the Illumina TruSeq stranded mRNA sample prep kit. The cDNA library was sequenced by paired‐end Illumina sequencing and analyzed as previously described.^[^ ^34^ ^]^

### Quantitative Real‐Time PCR

*S. aureus* USA300 was treated with celastrol (0.05 µg mL^−1^) and methicillin (25 µg mL^−1^) for 1 h at 37 °C. Total RNA was extracted using the Qiagen RNeasy kit. The RNA samples were used for cDNA synthesis using RNA to cDNA Ecodry Premix (Takara). Primers used for PCR are listed in Table S3 (Supporting Information). All Ct values were normalized to the respective Ct values of a housekeeping *gapA* gene^[^ ^34^ ^]^.

### Transmission Electron Microscopy of *S. aureus*

As previously described,^[^ ^23a^ ^]^ the overnight culture of *S. aureus* USA300 was inoculated in fresh media and grown to the exponential phase (OD_600_ of 0.4) at 37 °C. The bacteria were incubated in TSB containing 20 µm celastrol or DMSO at 37 °C for 30 min. After spinning down, a pellet of bacterial cells was fixed with 5% glutaraldehyde overnight. Fixed cells were washed with 0.05 m sodium cacodylate buffer three times and post‐fixed with 1% osmium tetroxide diluted in 0.1 m sodium cacodylate buffer for 2 h. The cells were washed with distilled water and then incubated in 0.5% uranyl acetate diluted in distilled water overnight. Cells were then washed with distilled water three times and dehydrated in ethanol with gradient from 30% to 100%. Subsequently, the bacterial cells were placed in propylene oxide and Spurr's resin mixture (1:1 v/v) for 1.5 h and incubated in 100% Spurr's resin overnight. Micrographs were taken using a FEI Tecnai F20 G2 transmission electron microscope.

### Statistical Analysis

All experiments were conducted three times with biological replicates. Results were presented as mean ± standard deviation (SD) or as indicated in each figure. Statistical analysis was performed using GraphPad Prism software version 8.0.2. An unpaired two‐tailed Student's t‐test was used to compare the means of two groups. Multiple comparisons were conducted using one‐way ANOVA followed by post hoc tests using Tukey's test. Statistical significance was defined as ^*^ *p* < 0.05, ^**^ *p* < 0.01, ^***^ *p* < 0.001, and ^****^ *p* < 0.0001.

## Conflict of Interest

The authors declare no conflict of interest.

## Author Contributions

W.L. conceived the project; W.L and S.J. designed and coordinated the overall study; J.K., Y.L., I.K., J.C., S.H., N.K.L., D.S., S.B. performed experiments; W.L., J.K., W.K., and S.J. performed the analysis. The manuscript was written by W.L. and edited by W.L., J.K., and J.C. with input from all authors.

## Supporting information

Supporting Information

## Data Availability

The data that support the findings of this study are available in the supplementary material of this article.
