# Supplementary material for: Reducing Peptidoglycan Crosslinking by Chemical Modulator Reverts β‐lactam Resistance in Methicillin‐Resistant Staphylococcus aureus
Source: Adv Sci (Weinh). 2024 May 15;11(28):2400858. doi: 10.1002/advs.202400858 (PMC11267302; doi:10.1002/advs.202400858)
Supplement: Supplementary file 1 — Supporting Information [file ADVS-11-2400858-s001.pdf]

ADVANCED SCIENCE
Open Access

Supporting Information

for *Adv. Sci.*, DOI 10.1002/advs.202400858

Reducing Peptidoglycan Crosslinking by Chemical Modulator Reverts β-lactam Resistance in Methicillin-Resistant *Staphylococcus aureus*

Ji-Hoon Kim, Yunmi Lee, Inseo Kim, JuOae Chang, Subin Hong, Na Kyung Lee, David Shum, Seongeun Baek, Wooseong Kim, Soojin Jang and Wonsik Lee**

Supporting Information

Reducing peptidoglycan crosslinking by chemical modulator reverts β-lactam resistance in methicillin-resistant *Staphylococcus aureus*

Ji-Hoon Kim[1], Yunmi Lee[2], Inseo Kim[1], JuOae Chang[1], Subin Hong[1], Na Kyung Lee[3], David Shum[3], Seongeun Baek[4], Wooseong Kim[4], Soojin Jang[2,*] and Wonsik Lee[1,*]

[1] School of Pharmacy, Sungkyunkwan University, Suwon 16419, Republic of Korea

[2] Antibacterial Resistance Laboratory, Institut Pasteur Korea, Seongnam 13488, Republic of Korea

[3] Screening Discovery Platform, Institut Pasteur Korea, Seongnam 13488, Republic of Korea

[4] College of Pharmacy, Graduate School of Pharmaceutical Sciences, Ewha Womans University, Seoul 03760, Republic of Korea

*Correspondence to: Wonsik Lee (wonsik.lee@skku.edu);
Sujin Jang (soojin.jang@ip-korea.org)

Contents	Page
Supporting Figures S1-S11	02-13
Supporting Table S1	14-21
Supporting Table S2	22
Supporting Table S3	23
Supporting References	24

class	>75% inhibition		
	compound	compound+met	number of compounds
I	X	X	2223
II	X	O	2
III	O	X	73
IV	O	O	23

Figure S1. Classification of screened compounds. The 2,321 compounds used for the screening were classified into four classes according to the bacterial growth inhibition profile. Growth inhibition was defined as more than 75% inhibition of bacterial growth compared to the control (not treated with any compound or drug).

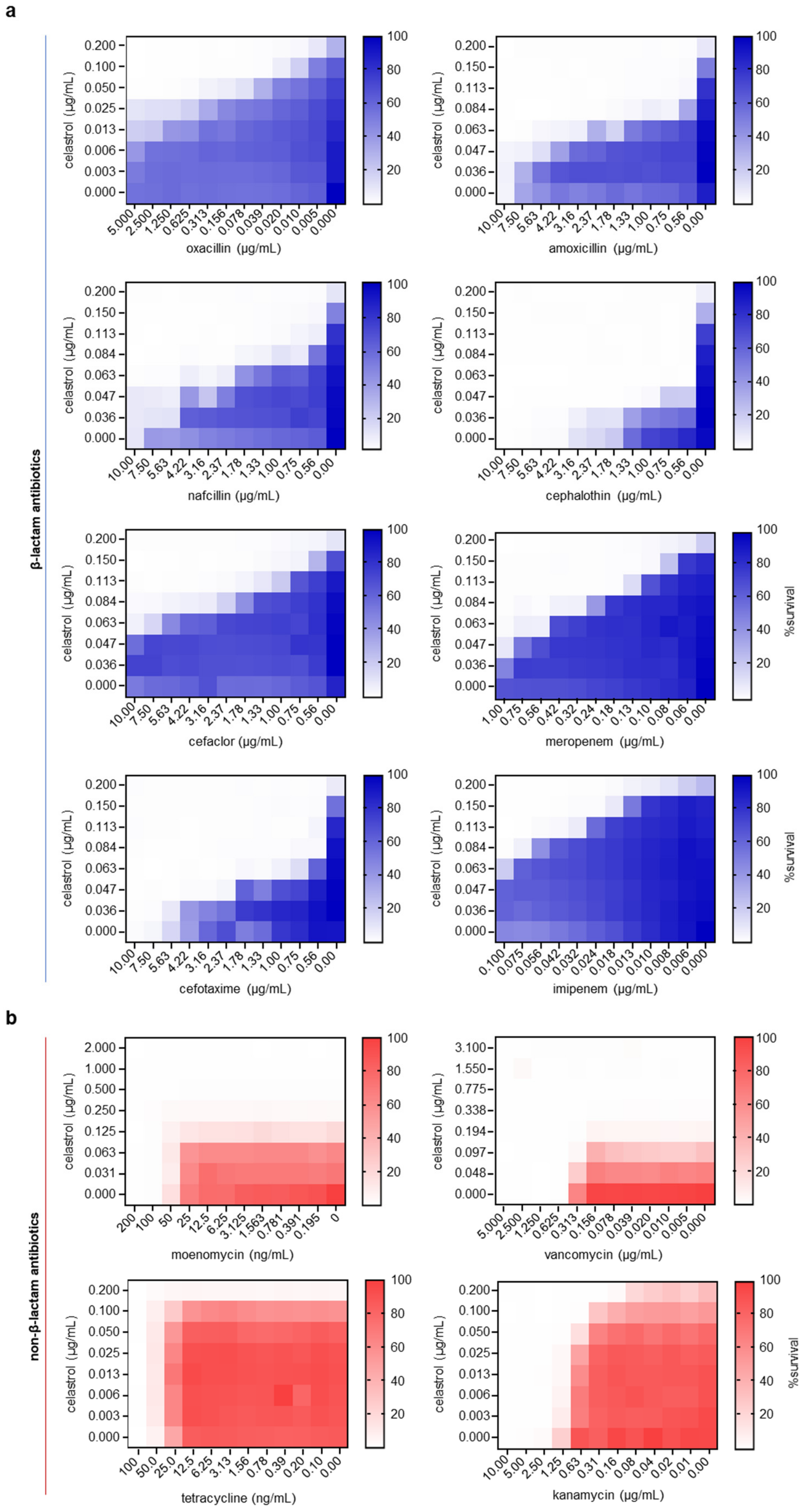
a
β-lactam antibiotics
celastrol (µg/mL)
0.200
0.100
0.050
0.025
0.013
0.006
0.003
0.000
5.000 2.500 1.250 0.625 0.313 0.156 0.078 0.039 0.020 0.010 0.005 0.000
oxacillin (µg/mL)
0.200 0.150 0.113 0.084 0.063 0.047 0.036 0.000
10.00 7.50 5.63 4.22 3.16 2.37 1.78 1.33 1.00 0.75 0.56 0.00
amoxicillin (µg/mL)
nafcillin (µg/mL)
cephalothin (µg/mL)
cefaclor (µg/mL)
1.00 0.75 0.56 0.42 0.32 0.24 0.18 0.13 0.10 0.08 0.06 0.00
meropenem (µg/mL)
cefotaxime (µg/mL)
0.100 0.075 0.056 0.042 0.032 0.024 0.018 0.013 0.010 0.008 0.006 0.000
imipenem (µg/mL)
100 80 60 40 20
%survival
b
non-β-lactam antibiotics
2.000 1.000 0.500 0.250 0.125 0.063 0.031 0.000
200 100 50 25 12.5 6.25 3.125 1.563 0.781 0.391 0.195 0
moenomycin (ng/mL)
3.100 1.550 0.775 0.338 0.194 0.097 0.048 0.000
5.000 2.500 1.250 0.625 0.313 0.156 0.078 0.039 0.020 0.010 0.005 0.000
vancomycin (µg/mL)
0.200 0.100 0.050 0.025 0.013 0.006 0.003 0.000
100 50.0 25.0 12.5 6.25 3.13 1.56 0.78 0.39 0.20 0.10 0.00
tetracycline (ng/mL)
10.00 5.00 2.50 1.25 0.63 0.31 0.16 0.08 0.04 0.02 0.01 0.00
kanamycin (µg/mL)

Figure S2. Checkerboard assay of combination of celastrol with β-lactam or non-β-lactam antibiotics in *S. aureus* USA300. a-b, USA300 cultured to log phase was used to confirm the function of celastrol as a re-sensitizer for β-lactam (**a**) or non-β-lactam (**b**) antibiotics using checkerboard assay. Bacterial growth was measured by OD_{600} using a plate reader upon treatment with combinations of various concentrations of celastrol and antibiotics. The results are representative of at least three independent experiments.

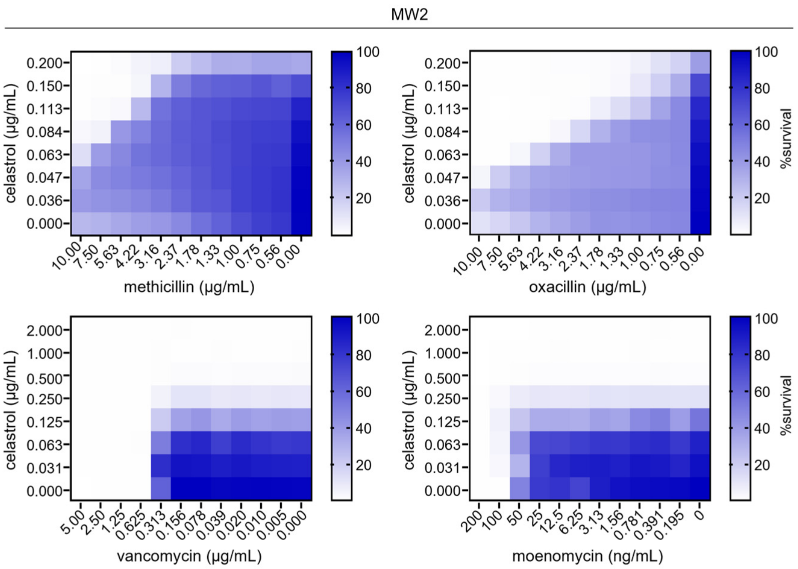

Figure S3. Restoration of β-lactam sensitivity by celastrol in MRSA strain MW2. Log phase culture of MRSA strain MW2 was used for validation of the role of celastrol as a re-sensitizer of β-lactam antibiotics using checkerboard assay. Bacterial growth was assessed by OD_{600} using a plate reader upon treatment with combinations of various concentrations of celastrol and antibiotics. The results are representative of at least three independent experiments.

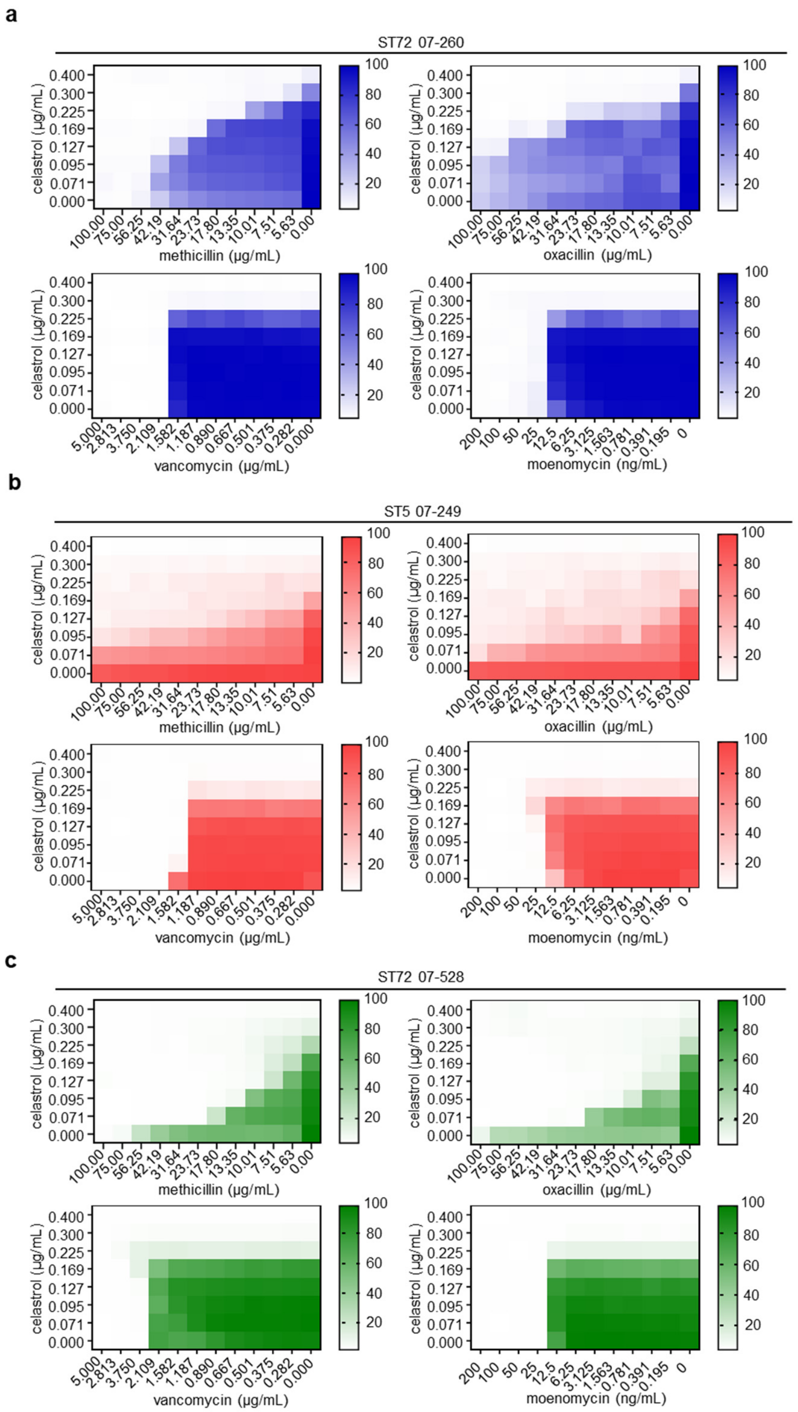

Figure S4. Reversal of β-lactam resistance in clinical MRSA strains. a-c, Cultures of clinical strains ST72 07-260 (**a**), ST5 07-249 (**b**) and ST72 07-528 (**c**) were grown to a log phase and used for validation of a role of celastrol in reversal of β-lactam resistance. Bacterial culture was grown under combinations of various concentrations of celastrol and antibiotics, and the growth was assessed by the OD_{600} values. The results are representative of at least three independent experiments.

a

strains	MIC (μg/mL)
S. aureus HG03	0.6
S. aureus USA300	0.6
S. aureus MW2	0.6
S. aureus MRSA252	0.6
Enterococcus faecalis (VRE)	0.3
Enterococcus faecalis (VSE)	0.3
Bacillus subtilis	0.15
Escherichia coli MG1655	>16
Pseudomonas aeruginosa PAO1	>16
Acinetobacter baumannii	>16

b

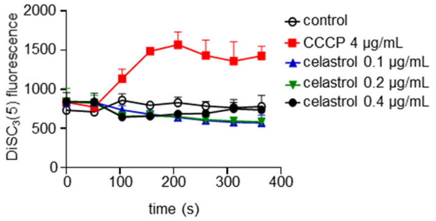

c

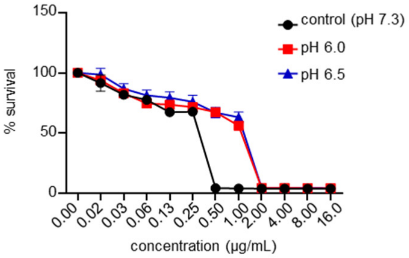

Figure S5. Antibacterial effect and PMF perturbation of celastrol in *S. aureus*. a, Celastrol MICs against a panel of Gram-positive and Gram-negative bacteria were determined. HG003 as a MSSA and USA300, MW2, and MRSA252 as MRSA strains were included. **b**, Fluorescence of DiSC$_{(3)}$5 dye was determined in CCCP or celastrol-treated USA300. **c**, MICs of celastrol against USA300 were determined at pH 6.0 or pH 6.5. Each data point represents mean ± SD from three biological replicates**.**

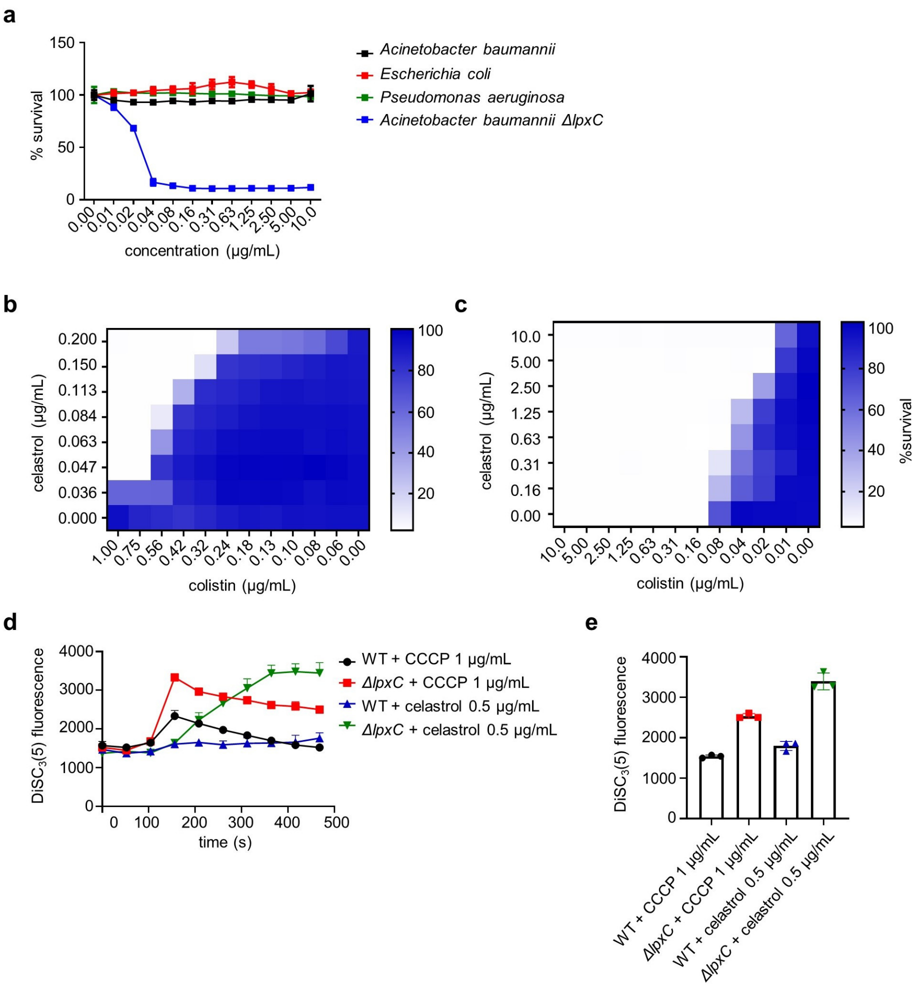

Figure S6. Effect of the outer membrane of Gram-negative bacteria on the activity of celastrol. **a**, The celastrol MICs were determined against Gram-negative strains by adding serial dilutions of celastrol concentrations ranging from 0.01 μg/mL to 10 μg/mL to bacterial cultures. **b-c**, The synergistic effect of celastrol and colistin was verified through a checkerboard assay in *A. baumannii* (**b**) and *E. coli* (**c**). The results are representative of at least three independent experiments. **d**, Fluorescence of $DiSC_{(3)}5$ dye was determined in CCCP or celastrol-treated *A. baumannii* WT or Δ*lpxC*. **e**, Fluorescence intensity of $DiSC_{(3)}5$ dye after 500 seconds of drug treatment was graphed. The values are in mean ± SD of three biological replicates.

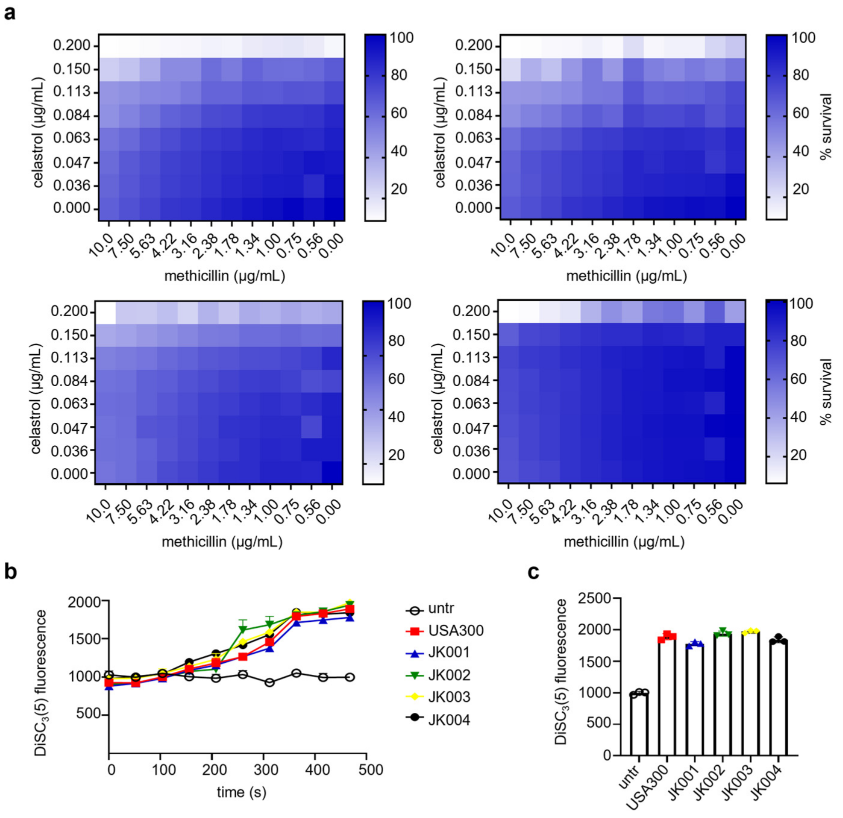

Figure S7. Verification of the role of celastrol as a β-lactam re-sensitizer and PMF perturbator in JK001-004. a. The methicillin re-sensitization effect of celastrol was measured with checkerboard assay in JK001-004 strains. The results are representative of at least three independent experiments. **b**. *S. aureus* USA300 and JK001-004 strains were treated with 8 µg/mL celastrol, and PMF perturbation was determined by $DiSC_{(3)}5$ dye. **c**. $DiSC_{(3)}5$ fluorescence intensity after 500 seconds of celastrol treatment was represented as a bar graph. The values are in mean ± SD of three biological replicates.

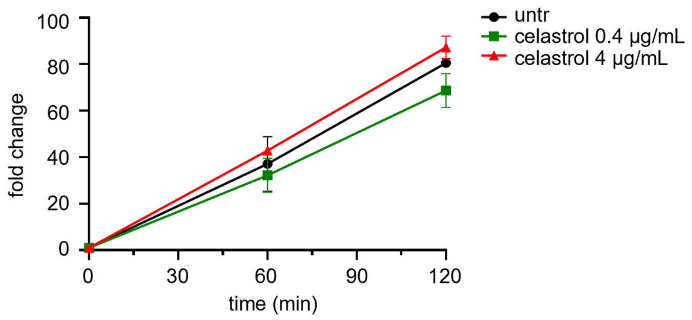

Figure S8. Regulation of DacA activity by celastrol. ATP, substrate for DacA, was added to the reaction buffer containing DacA and DMSO (untr) or 0.4 or 4 µg/mL celastrol. The amount of c-di-AMP produced by the reaction was measured by LC-ToF/MS at various time points post-addition of ATP. The values are in mean ± SD of three biological replicates.

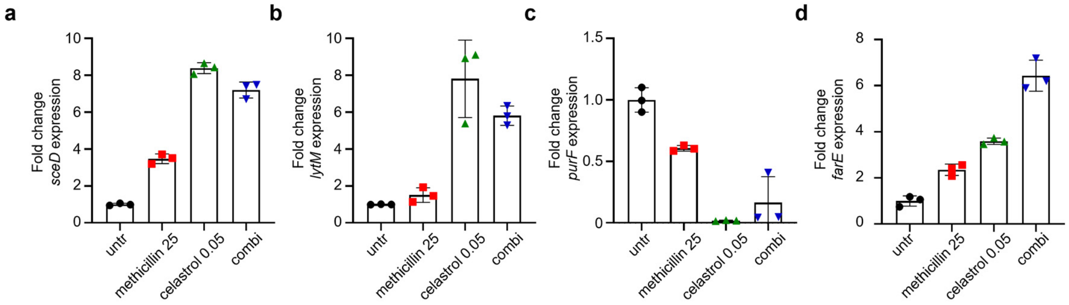

Figure S9. Changes in gene expressions of USA300 in the presence of drugs. a-d, Changes in expressions of *sceD* (**a**), *lytM* (**b**), *purF* (**c**), and *farE* (**d**) 1 hour post- treatment with methicillin 25 µg/mL, celastrol 0.05 µg/mL, or combination of both were analyzed by quantitative PCR. The expression values were normalized to *gapA* used as an internal control. The values are in mean ± SD of three biological replicates.

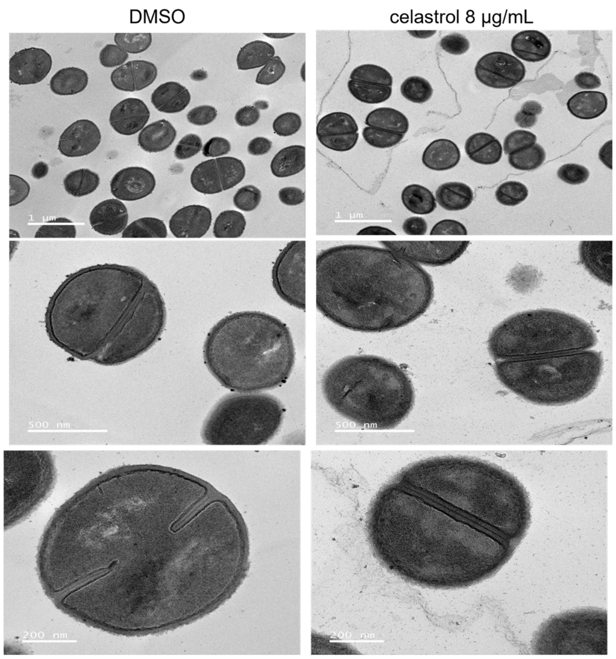

Figure S10. Transmission electron microscopy images of *S. aureus* USA300 treated with celastrol. Morphological changes in septa and cytoplasm of USA300 were examined after treatment with 8 µg/mL celastrol for 30 min at 37°C. The scale bar (200 nm) is presented on the left of the images.

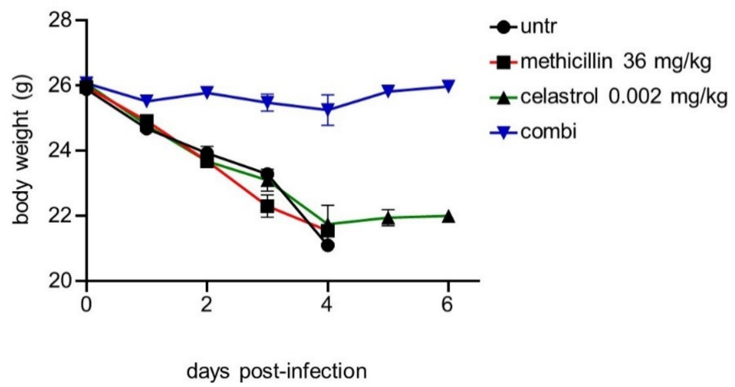

Figure S11. Body weights of mice. Mice infected with USA300 were divided into four groups (14 mice/group): 1) mice not treated with any drug (untreated), 2) mice treated with methicillin 36 mg/kg, 3) mice treated with celastrol 0.002 mg/kg, and 4) mice treated with a combination of methicillin (36 mg/kg) and celastrol (0.002 mg/kg). Body weights of the animals were measured over 6 days post-infection. The values are in mean ± SEM.

Table S1. Gene expression analysis for the celastrol-treated USA300

locus_tag	Name	Fold change (log2)	p-Value
SAUSA300_2489	*farE*	11.09	9.563E-259
SAUSA300_2493	*cwrA*	4.84	1.406E-79
SAUSA300_0085	*cstA*	4.57	2.488E-39
SAUSA300_0207	.	3.97	1.066E-40
SAUSA300_0432	.	3.46	6.762E-49
SAUSA300_2414	*cntK*	3.31	4.508E-32
SAUSA300_1606	.	3.28	3.281E-42
SAUSA300_2412	*cntM*	3.27	3.078E-34
SAUSA300_2051	*sceD*	3.13	1.591E-32
SAUSA300_2413	*cntL*	2.98	6.597E-24
SAUSA300_1193	*glpD*	2.93	2.100E-40
SAUSA300_1092	*pyrP*	2.93	3.689E-40
SAUSA300_2616	*ecfT*	2.87	7.778E-27
SAUSA300_0086	*cstB*	2.87	2.711E-29
SAUSA300_0622	.	2.76	7.859E-31
SAUSA300_1091	*pyrR*	2.75	6.511E-36
SAUSA300_0093	.	2.71	1.602E-16
SAUSA300_1706	.	2.62	1.178E-31
SAUSA300_1374	.	2.60	3.001E-25
SAUSA300_2302	*tcaA*	2.60	1.497E-32
SAUSA300_0777	*cspC*	2.60	7.368E-33
SAUSA300_0188	*brnQ*	2.55	5.054E-30
SAUSA300_0863	*argH*	2.51	1.463E-28
SAUSA300_0319	.	2.51	1.448E-29
SAUSA300_2304	.	2.43	5.633E-26
SAUSA300_1073	*mraW*	2.43	3.622E-26
SAUSA300_0864	*argG*	2.41	1.511E-26
SAUSA300_2546	*betB*	2.38	7.746E-25
SAUSA300_0867	*lepB*	2.35	7.294E-26
SAUSA300_0228	*fadE*	2.34	1.723E-18
SAUSA300_1093	*pyrB*	2.32	5.213E-27
SAUSA300_1326	.	2.30	7.028E-13
SAUSA300_2578	.	2.29	1.899E-17
SAUSA300_0629	*pbp4*	2.25	1.198E-23
SAUSA300_1583	.	2.24	4.271E-14
SAUSA300_0229	.	2.22	1.245E-03
SAUSA300_1882	*gatC*	2.21	1.105E-21
SAUSA300_1796	.	2.21	6.287E-22
SAUSA300_1089	*lspA*	2.18	7.670E-18
SAUSA300_1609	.	2.14	1.032E-02
SAUSA300_0089	.	2.13	1.004E-11

Table S1. continued			
locus_tag	Name	Fold change (log2)	p-Value
SAUSA300_1416	.	2.13	6.630E-03
SAUSA300_1865	*vraR*	2.11	1.598E-22
SAUSA300_1867	*liaF*	2.09	1.888E-20
SAUSA300_2350	.	2.07	1.953E-15
SAUSA300_0697	*queC*	2.06	2.283E-17
SAUSA300_2262	*sdpB*	2.06	9.921E-22
SAUSA300_2440	*fnbB*	2.05	8.625E-17
SAUSA300_1094	*pyrC*	2.03	1.560E-21
SAUSA300_1855	*sgtB*	2.02	5.863E-21
SAUSA300_0866	.	2.00	1.317E-18
SAUSA300_2490	*farR*	2.00	3.173E-19
SAUSA300_1588	*lytH*	2.00	2.947E-20
SAUSA300_2441	*fnbA*	1.98	3.428E-16
SAUSA300_0959	*fmtA*	1.96	7.302E-20
SAUSA300_2372	*bioA*	1.96	5.991E-10
SAUSA300_1097	*pyrF*	1.95	6.097E-20
SAUSA300_0117	*sirA*	1.95	8.976E-19
SAUSA300_0986	.	1.94	2.482E-19
SAUSA300_1154	*cdsA*	1.94	2.003E-19
SAUSA300_0902	*pepF*	1.94	7.480E-20
SAUSA300_2046	*yidC*	1.92	1.314E-19
SAUSA300_0914	.	1.92	2.017E-19
SAUSA300_0831	.	1.92	3.380E-06
SAUSA300_2291	*gltS*	1.92	1.963E-14
SAUSA300_0848	.	1.91	9.916E-08
SAUSA300_1098	*pyrE*	1.90	6.333E-19
SAUSA300_1283	*gid*	1.89	5.425E-19
SAUSA300_1283	*pstS*	1.89	2.123E-04
SAUSA300_2609	*hisB*	1.88	1.086E-04
SAUSA300_1068	*psmB2*	1.88	8.835E-04
SAUSA300_2144	.	1.86	5.127E-11
SAUSA300_2373	*bioD*	1.85	1.534E-04
SAUSA300_1096	*carB*	1.84	2.555E-18
SAUSA300_1371	*recQ*	1.84	9.914E-18
SAUSA300_1095	*carA*	1.84	3.621E-18
SAUSA300_0931	.	1.81	3.114E-14
SAUSA300_0270	*lytM*	1.80	4.976E-17
SAUSA300_1192	*glpK*	1.80	1.120E-16
SAUSA300_2565	*clfB*	1.80	4.061E-17
SAUSA300_0703	*ltaS*	1.79	1.876E-17
SAUSA300_2545	*betA*	1.79	1.071E-15
SAUSA300_0163	.	1.79	8.781E-04

**Table S1. continued**			
**locus_tag**	**Name**	**Fold change (log2)**	**p-Value**
SAUSA300_2299	.	1.79	1.084E-11
SAUSA300_0443	.	1.79	5.038E-16
SAUSA300_1191	*glpF*	1.79	7.341E-16
SAUSA300_2492	.	1.78	2.007E-15
SAUSA300_0085	.	1.77	1.616E-09
SAUSA300_0992	.	1.76	2.067E-16
SAUSA300_1143	*topA*	1.76	8.899E-17
SAUSA300_0442	.	1.75	6.874E-16
SAUSA300_2139	*sfaA*	1.74	3.486E-14
SAUSA300_0773	*vwb*	1.73	1.170E-04
SAUSA300_2298	.	1.72	3.601E-15
SAUSA300_1998	.	1.71	7.247E-14
SAUSA300_1134	*rplS*	1.67	1.803E-15
SAUSA300_0704	.	1.66	1.094E-14
SAUSA300_1570	.	1.65	6.699E-15
SAUSA300_0719	.	1.65	1.133E-13
SAUSA300_1172	.	1.65	3.111E-14
SAUSA300_0107	.	1.64	8.891E-15
SAUSA300_2603	*lip*	1.64	2.584E-12
SAUSA300_0877	*clpB*	1.63	8.702E-15
SAUSA300_2521	.	1.63	2.483E-06
SAUSA300_0987	.	1.63	1.222E-13
SAUSA300_2601	*icaB*	1.62	1.917E-02
SAUSA300_0224	*coa*	1.62	2.099E-06
SAUSA300_1568	*udk*	1.61	5.091E-14
SAUSA300_1013	*ftsW*	1.61	3.663E-14
SAUSA300_0985		1.60	2.604E-06
SAUSA300_2233	.	1.60	7.187E-12
SAUSA300_1113	*pknB*	1.55	1.701E-13
SAUSA300_2607	*hisA*	1.55	9.456E-04
SAUSA300_1730	*metK*	1.55	2.271E-13
SAUSA300_0741	*uvrB*	1.55	2.395E-13
SAUSA300_2078	*murA*	1.54	2.258E-13
SAUSA300_1569	.	1.54	7.744E-13
SAUSA300_1517	.	1.54	5.253E-13
SAUSA300_2610	.	1.53	6.213E-06
SAUSA300_0654	*sarX*	1.50	6.453E-04
SAUSA300_0509	.	1.50	9.136E-13
SAUSA300_0972	*purF*	-7.24	9.080E-156
SAUSA300_0974	*purN*	-7.23	1.709E-147
SAUSA300_0971	*purL*	-7.20	6.327E-156
SAUSA300_0973	*purM*	-7.10	9.215E-149

Table S1. continued			
locus_tag	Name	Fold change (log2)	p-Value
SAUSA300_0975	*purH*	-7.09	1.931E-152
SAUSA300_0970	*purQ*	-6.97	6.515E-142
SAUSA300_0151	*adhE*	-6.94	1.198E-147
SAUSA300_0220	*pflB*	-6.89	1.346E-149
SAUSA300_0221	*pflA*	-6.81	7.341E-141
SAUSA300_0976	*purD*	-6.68	8.690E-141
SAUSA300_0968	*purC*	-6.60	5.060E-125
SAUSA300_0966	*purE*	-6.50	5.615E-110
SAUSA300_0967	*purK*	-6.47	6.583E-132
SAUSA300_1331	*ald*	-6.44	4.368E-85
SAUSA300_0969	*purS*	-6.38	6.676E-85
SAUSA300_0257	*lrgB*	-5.94	1.585E-114
SAUSA300_1330	*tdcB*	-5.88	4.741E-78
SAUSA300_0256	*lrgA*	-5.88	1.846E-99
SAUSA300_0108	.	-5.58	3.402E-97
SAUSA300_0594	*adhP*	-5.37	7.297E-103
SAUSA300_2572	*aur*	-5.31	2.493E-103
SAUSA300_0949	*sspC*	-5.31	1.645E-89
SAUSA300_0951	*sspA*	-5.10	2.150E-95
SAUSA300_0950	*sspB*	-5.05	3.280E-94
SAUSA300_2343	.	-4.85	4.921E-86
SAUSA300_2486	*clpL*	-4.79	6.935E-87
SAUSA300_2346	*nirB*	-4.36	4.438E-71
SAUSA300_0232	.	-4.24	2.003E-52
SAUSA300_0769	.	-4.23	2.386E-54
SAUSA300_1755	*splD*	-4.15	2.222E-24
SAUSA300_2347	.	-4.13	3.835E-58
SAUSA300_2342	*narH*	-4.13	1.807E-51
SAUSA300_1922	*sak*	-4.11	1.189E-66
SAUSA300_2333	.	-4.11	5.874E-64
SAUSA300_0234	.	-4.08	1.983E-69
SAUSA300_1758	*splA*	-3.77	7.979E-24
SAUSA300_0253	*scdA*	-3.68	6.197E-53
SAUSA300_1668	.	-3.68	1.507E-49
SAUSA300_0278	*esxA*	-3.63	1.119E-49
SAUSA300_1756	*splC*	-3.56	7.738E-29
SAUSA300_1974	*lukG*	-3.55	2.001E-49
SAUSA300_0282	*essB*	-3.51	5.960E-36
SAUSA300_1975	*lukH*	-3.49	3.995E-48
SAUSA300_1757	*splB*	-3.49	1.496E-24
SAUSA300_2418	.	-3.46	1.912E-47
SAUSA300_0099	.	-3.45	5.872E-37

Table S1. continued			
**locus_tag**	**Name**	**Fold change (log2)**	**p-Value**
SAUSA300_2525	.	-3.42	3.346E-44
SAUSA300_2345	*nirD*	-3.40	1.028E-21
SAUSA300_2500	*crtQ*	-3.38	1.728E-41
SAUSA300_0017	*purA*	-3.28	1.194E-48
SAUSA300_2502	*crtO*	-3.25	2.956E-25
SAUSA300_0285	*esxB*	-3.24	3.813E-17
SAUSA300_1474	.	-3.23	1.372E-47
SAUSA300_1445	*scpA*	-3.18	7.653E-42
SAUSA300_0286	.	-3.16	6.128E-29
SAUSA300_1564	.	-3.16	8.412E-14
SAUSA300_1759	.	-3.16	1.367E-35
SAUSA300_1475	.	-3.10	1.113E-33
SAUSA300_0283	*essC*	-3.10	1.290E-40
SAUSA300_1711	.	-3.07	6.746E-32
SAUSA300_2641	.	-3.05	4.999E-05
SAUSA300_0235	.	-3.05	2.714E-43
SAUSA300_2632	.	-3.04	6.832E-37
SAUSA300_2573	*isaB*	-3.04	1.733E-38
SAUSA300_1562	*pxpA*	-3.01	9.179E-28
SAUSA300_0263	*rbsD*	-2.98	2.803E-34
SAUSA300_0264	*rbsU*	-2.96	3.397E-38
SAUSA300_1456	.	-2.95	8.354E-38
SAUSA300_0793	.	-2.92	8.067E-38
SAUSA300_2144	*amaP*	-2.91	6.135E-40
SAUSA300_2281	*hutG*	-2.89	3.793E-38
SAUSA300_0262	*rbsK*	-2.88	8.327E-37
SAUSA300_0280	*essA*	-2.85	2.394E-13
SAUSA300_2465	*cobA*	-2.85	1.588E-31
SAUSA300_0816	.	-2.84	3.167E-38
SAUSA300_1754	*splE*	-2.81	2.891E-17
SAUSA300_1852	.	-2.81	1.076E-25
SAUSA300_0965	*folD*	-2.79	4.750E-37
SAUSA300_2498	.	-2.78	6.542E-30
SAUSA300_2396	.	-2.78	2.123E-35
SAUSA300_0374	.	-2.76	3.184E-36
SAUSA300_0284	*esaC*	-2.76	1.300E-09
SAUSA300_0320	*lip2*	-2.76	4.587E-36
SAUSA300_1753	*splF*	-2.76	1.054E-15
SAUSA300_0736	*raiA*	-2.74	6.897E-36
SAUSA300_2539	.	-2.71	4.492E-26
SAUSA300_0025	*adsA*	-2.71	2.919E-31
SAUSA300_2327	.	-2.70	1.297E-33

**Table S1. continued**			
**locus_tag**	**Name**	**Fold change (log2)**	**p-Value**
SAUSA300_0794	.	-2.68	6.883E-29
SAUSA300_2537	.	-2.65	6.343E-34
SAUSA300_1917	*eap*	-2.65	9.735E-28
SAUSA300_1714	*ribE*	-2.64	1.372E-32
SAUSA300_0637	*dhaL*	-2.64	5.129E-17
SAUSA300_2619	.	-2.61	1.963E-09
SAUSA300_0332	.	-2.60	5.176E-25
SAUSA300_1761	.	-2.60	2.373E-21
SAUSA300_1065	.	-2.59	7.291E-30
SAUSA300_0424.3	.	-2.58	2.512E-10
SAUSA300_0279	*esaA*	-2.56	1.285E-29
SAUSA300_0257	.	-2.56	2.865E-30
SAUSA300_1235	*guaC*	-2.55	1.976E-31
SAUSA300_0183	.	-2.52	4.433E-31
SAUSA300_1714	*ribB*	-2.50	1.097E-30
SAUSA300_2398	*fetB*	-2.50	3.206E-29
SAUSA300_0192	.	-2.47	1.057E-22
SAUSA300_2626	*bstA*	-2.44	7.999E-26
SAUSA300_0136	*sasD*	-2.43	7.756E-29
SAUSA300_1306	.	-2.42	5.161E-29
SAUSA300_0311	*pfkB*	-2.41	6.838E-29
SAUSA300_1714	.	-2.40	1.789E-28
SAUSA300_0193	*murQ*	-2.38	2.865E-17
SAUSA300_0636	*dhaK*	-2.37	1.154E-22
SAUSA300_2108	.	-2.35	3.319E-26
SAUSA300_1769	*lukE*	-2.31	2.399E-08
SAUSA300_1874	*ftnA*	-2.29	6.960E-26
SAUSA300_2114	*rocF*	-2.27	4.972E-24
SAUSA300_2289	.	-2.23	3.381E-22
SAUSA300_2006	*ilvD*	-2.22	2.590E-21
SAUSA300_1715	*ribD*	-2.22	4.528E-25
SAUSA300_1305	*sucB*	-2.21	1.369E-24
SAUSA300_2156	.	-2.20	9.429E-25
SAUSA300_1768	*lukD*	-2.16	2.433E-09
SAUSA300_0008	*hutH*	-2.14	5.415E-18
SAUSA300_1599	.	-2.14	4.035E-06
SAUSA300_1246	*acnA*	-2.13	1.395E-23
SAUSA300_1678	.	-2.13	1.404E-23
SAUSA300_2477	.	-2.13	3.605E-23
SAUSA300_0307	.	-2.11	1.928E-22
SAUSA300_0701	*pxpB*	-2.10	2.836E-12
SAUSA300_2589	*sasA*	-2.07	1.813E-21

**Table S1. continued**			
**locus_tag**	**Name**	**Fold change (log2)**	**p-Value**
SAUSA300_2324	.	-2.05	1.763E-21
SAUSA300_1498	*gcvT*	-2.05	1.867E-21
SAUSA300_0772	*clfA*	-2.05	6.169E-22
SAUSA300_0756	.	-2.02	1.506E-16
SAUSA300_1655	*ald*	-1.98	2.545E-20
SAUSA300_0475	*spoVG*	-1.98	1.722E-20
SAUSA300_2443	*gntK*	-1.98	1.459E-17
SAUSA300_0940	.	-1.96	2.283E-19
SAUSA300_0638	*dhaM*	-1.95	1.064E-13
SAUSA300_1976	.	-1.95	1.496E-17
SAUSA300_0474	.	-1.95	5.712E-20
SAUSA300_1311	.	-1.93	2.243E-19
SAUSA300_2641	.	-1.93	2.011E-10
SAUSA300_1381	*lukF-PV*	-1.91	2.337E-18
SAUSA300_2161	*hysA*	-1.91	2.246E-18
SAUSA300_1382	*lukS-PV*	-1.87	8.452E-18
SAUSA300_0113	*spa*	-1.87	1.082E-10
SAUSA300_0936	.	-1.87	4.218E-14
SAUSA300_2007	*ilvB*	-1.86	1.091E-14
SAUSA300_0145	*phnD*	-1.84	3.390E-08
SAUSA300_0245	.	-1.84	2.363E-17
SAUSA300_0360	*metI*	-1.83	5.907E-10
SAUSA300_0379	*ahpF*	-1.80	1.400E-17
SAUSA300_0329	.	-1.80	3.363E-17
SAUSA300_0380	*ahpC*	-1.79	2.337E-17
SAUSA300_1879	.	-1.77	3.303E-16
SAUSA300_1864	.	-1.76	8.620E-17
SAUSA300_2207	.	-1.76	8.067E-17
SAUSA300_1765	.	-1.75	1.749E-07
SAUSA300_1497	*gcvPA*	-1.74	1.667E-16
SAUSA300_1442	*srrA*	-1.74	1.928E-16
SAUSA300_0364	*fdhF*	-1.73	2.485E-16
SAUSA300_2257	.	-1.68	8.051E-14
SAUSA300_0129	.	-1.67	5.761E-15
SAUSA300_0297	.	-1.67	7.488E-11
SAUSA300_0242	*gutB*	-1.66	9.890E-15
SAUSA300_2328	.	-1.66	6.545E-15
SAUSA300_2540	.	-1.65	3.590E-15
SAUSA300_1925	.	-1.65	1.375E-06
SAUSA300_0196	*hsdR*	-1.64	1.783E-14
SAUSA300_2104	*glmS*	-1.64	5.997E-15
SAUSA300_0288	.	-1.62	9.051E-14

**Table S1. continued**			
**locus_tag**	**Name**	**Fold change (log2)**	**p-Value**
SAUSA300_0197	.	-1.60	4.536E-13
SAUSA300_0424.4	*psmα1*	-1.60	5.464E-03
SAUSA300_0315	*nanA*	-1.60	2.141E-04
SAUSA300_0448	*treP*	-1.59	4.991E-14
SAUSA300_2340	*narI*	-1.59	2.573E-10
SAUSA300_0386	*xpt*	-1.59	9.467E-13
SAUSA300_2442	*gntP*	-1.57	3.152E-12
SAUSA300_0584	.	-1.57	2.886E-07
SAUSA300_0042	*aaa*	-1.56	1.183E-13
SAUSA300_2364	*sbi*	-1.56	1.387E-13
SAUSA300_1801	*fumC*	-1.56	1.373E-13
SAUSA300_1862	.	-1.56	2.165E-13
SAUSA300_1496	*gcvPB*	-1.55	1.373E-13
SAUSA300_2618	.	-1.55	3.166E-06
SAUSA300_0410	*lpl1*	-1.53	4.910E-05
SAUSA300_1809	.	-1.52	4.343E-10
SAUSA300_2285	.	-1.52	2.259E-12
SAUSA300_2090	*deoC*	-1.51	2.830E-12

Table S2. Bacterial strains used in this study

Strain	Relevant features	Source or reference
S. aureus HG003	Wild-type	Dr. Suzanne Walker
S. aureus USA300	Wild-type	Dr. Suzanne Walker
S. aureus USA300 pLOW-*gdpP*	*S. aureus* USA300 with pLOW-*gdpP* containing *ftsZ* RBS	This study
S. aureus USA300 *ΔgdpP*	*S. aureus* USA300 *ΔgdpP::*Spec^R	This study
S. aureus USA300 pET-28a-*gdpP*	*S. aureus* USA300 with pET-28a: His6-GdpP	1
S. aureus USA300 pET-28a-*dacA*	*S. aureus* USA300 with pET-28a: His6-DacA	2
S. aureus USA300 JK001-004	Spontaneous mutants	This study
S. aureus USA300 Tn::*lytM*	Transposon insertion in SAUSA300_0270	3
S. aureus USA300 ST72 07-260	Clinical isolate	Dr. Kwan Soo Ko
S. aureus USA300 ST5 07-249	Clinical isolate	Dr. Kwan Soo Ko
S. aureus USA300 ST72 07-528	Clinical isolate	Dr. Kwan Soo Ko
S. aureus MW2	Wild-type	Dr. Suzanne Walker
S. aureus RN4220 *ΔgdpP*	*S. aureus* RN4220 *ΔgdpP::*Spec^R	4
E. faecalis (VRE)	Wild-type	Dr. Suzanne Walker
E. faecalis (VSE)	Wild-type	Dr. Suzanne Walker
B. subtilis	Wild-type	Dr. Suzanne Walker
P. aeruginosa PAO1	Wild-type	Dr. Suzanne Walker
A. baumannii	Wild-type	Dr. Suzanne Walker
A. baumannii ΔlpxC	*A. baumannii ΔlpxC*	5
E. coli BL21(DE3)	Expression strain for protein production	6
E. coli DH5α pET-28a	*E. coli* DH5α with pET-28a, IPTG-inducible protein expression vector; Kan^R	Novagen
E. coli DH5α pET-28a-*gdpP*	*E. coli* DH5α with pET-28a: His6-GdpP	1
E. coli DH5α pET-28a-*dacA*	*E. coli* DH5α with pET-28a: His6-DacA	2
E. coli DH5α pLOW	*E. coli* DH5α with pLOW, *S. aureus* expression vector containing IPTG-inducible P_{spac} promoter; Erm^R	7
E. coli DH5α pLOW-*gdpP*	*E. coli* DH5α with pLOW-*gdpP* containing *ftsZ* RBS	This study

*Abbreviations: Spec^R, spectinomycin resistance; Kan^R, kanamycin resistance; Erm^R, erythromycin resistance.

Table S3. Primers used in this study

primer	Sequence 5'-3'
*lytM*_F	CACAGCGAGTCAAAGCCAAC
*lytM*_R	TGATTGCCGCCACCATAGTT
*sceD*_F	GAAATGCAGGTCACGAAGC
*sceD*_R	AAGCCCCAGCTTCAATTGGT
*purF*_F	CATGCAATTGGTCACGTCCG
*purF*_R	GCATTTGGATCTACTGCGCC
*farE*_F	CCGCGGTCATTGCATGGATA
*farE*_R	TGCAGCAATGACTGAACCGA
*GAPDH*_F	TGCAAGGTCGTTTCACAGGT
*GAPDH*_R	GGGATGATGTTTTCTGCCGC

1. Corrigan, R.M., Abbott, J.C., Burhenne, H., Kaever, V. & Grundling, A. c-di-AMP is a new second messenger in *Staphylococcus aureus* with a role in controlling cell size and envelope stress. *PLoS Pathog* **7**, e1002217 (2011).
2. Tosi, T. et al. Inhibition of the *Staphylococcus aureus* c-di-AMP cyclase DacA by direct interaction with the phosphoglucosamine mutase GlmM. *PLoS Pathog* **15**, e1007537 (2019).
3. Fey, P.D. et al. A genetic resource for rapid and comprehensive phenotype screening of nonessential *Staphylococcus aureus* genes. *mBio* **4**, e00537-12 (2013).
4. Pasquina, L. et al. A synthetic lethal approach for compound and target identification in *Staphylococcus aureus*. *Nat Chem Biol* **12**, 40-5 (2016).
5. Lee, W. et al. Antibiotic combinations that enable one-step, targeted mutagenesis of chromosomal genes. *ACS Infect Dis* **4**, 1007-1018 (2018).
6. Studier, F.W. & Moffatt, B.A. Use of bacteriophage T7 RNA polymerase to direct selective high-level expression of cloned genes. *J Mol Biol* **189**, 113-30 (1986).
7. Liew, A.T.F. et al. A simple plasmid-based system that allows rapid generation of tightly controlled gene expression in *Staphylococcus aureus*. *Microbiology (Reading)* **157**, 666-676 (2011).
